# Systematic Discovery of Endogenous Human Ribonucleoprotein Complexes

**DOI:** 10.1016/j.celrep.2019.09.060

**Published:** 2019-10-29

**Authors:** Anna L. Mallam, Wisath Sae-Lee, Jeffrey M. Schaub, Fan Tu, Anna Battenhouse, Yu Jin Jang, Jonghwan Kim, John B. Wallingford, Ilya J. Finkelstein, Edward M. Marcotte, Kevin Drew

**Affiliations:** 1 Department of Molecular Biosciences, University of Texas at Austin, Austin, TX 78712, USA; 2 Center for Systems and Synthetic Biology, University of Texas at Austin, Austin, TX 78712, USA; 3 Institute for Cellular and Molecular Biology, University of Texas at Austin, Austin, TX 78712, USA; 4 These authors contributed equally; 5 Lead Contact

## Abstract

RNA-binding proteins (RBPs) play essential roles in biology and are frequently associated with human disease. Although recent studies have systematically identified individual RNA-binding proteins, their higher-order assembly into ribonucleoprotein (RNP) complexes has not been systematically investigated. Here, we describe a proteomics method for systematic identification of RNP complexes in human cells. We identify 1,428 protein complexes that associate with RNA, indicating that more than 20% of known human protein complexes contain RNA. To explore the role of RNA in the assembly of each complex, we identify complexes that dissociate, change composition, or form stable protein-only complexes in the absence of RNA. We use our method to systematically identify cell-type-specific RNA-associated proteins in mouse embryonic stem cells and finally, distribute our resource, rna.MAP, in an easy-to-use online interface (rna.proteincomplexes.org). Our system thus provides a methodology for explorations across human tissues, disease states, and throughout all domains of life.

## In Brief

Ribonucleoprotein (RNP) complexes carry out many essential biological processes. Mallam et al. developed differential fractionation (DIF-FRAC), a proteomics method to systematically discover RNP complexes. Using their method, they discovered previously unknown RNP complexes, classified complexes by their RNA-dependent stability, and identified previously unknown roles for RNA in known protein complexes.

## Graphical Abstract

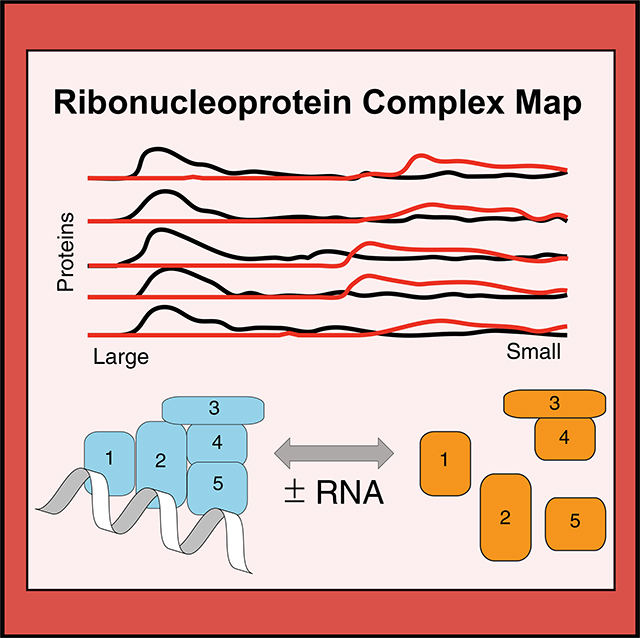

## INTRODUCTION

RNA-binding proteins (RBPs) play essential roles in diverse biological processes and in most cases act within higher order multi-protein complexes called ribonucleoprotein (RNP) complexes (Castello et al., 2013; Gerstberger et al., 2014; Hentze et al., 2018). Understanding RNPs is of particular importance because of their indispensable role in many essential cellular functions, such as mRNA splicing (spliceosome) (Wahl et al., 2009), translation (ribosome) (Ramakrishnan, 2002), gene silencing (Kawamata and Tomari, 2010), and degradation (exosome) (Houseley et al., 2006). Moreover, RNPs also play more specific roles in, for example, mRNA transport and localization in developing embryos and mature neurons (Holt and Bullock, 2009; Sahoo et al., 2018) and assembly of phase separated organelles (Mittag and Parker, 2018). Furthermore, RNPs are strongly implicated in human diseases including amyotrophic lateral sclerosis (ALS) (Scotter et al., 2015), spinocerebellar ataxia (Yue et al., 2001), and autism (Voineagu et al., 2011). Accordingly, substantial recent effort has been focused on systematic identification of RNA-binding proteins (Baltz et al., 2012; Bao et al., 2018; Beckmann et al., 2015; Brannan et al., 2016; Castello et al., 2012, 2016; Caudron-Herger et al., 2019; Conrad et al., 2016; He et al., 2016; Huang et al., 2018; Kramer et al., 2014; Queiroz et al., 2019; Treiber et al., 2017; Trendel et al., 2019).

Strikingly, however, we still lack any systematic characterization of the assembly of individual proteins which associate with RNA in higher order RNP complexes, leaving a crucial gap in our knowledge. Here we define an RNA-associated protein as a protein that physically interacts directly with RNA or indirectly with RNA through a secondary interacting molecule. A worldwide effort is currently under way to systematically identify multi-protein complexes using high-throughput mass spectrometry techniques (Hein et al., 2015; Huttlin et al., 2015), but none of these techniques identify an RNA component within the complexes. We therefore set out to develop a method for systematic identification of RNA-associated higher order multi-protein complexes that requires no genetic manipulation (i.e., tag-free) and would be easily adaptable to diverse cell types.

**Figure 1.**
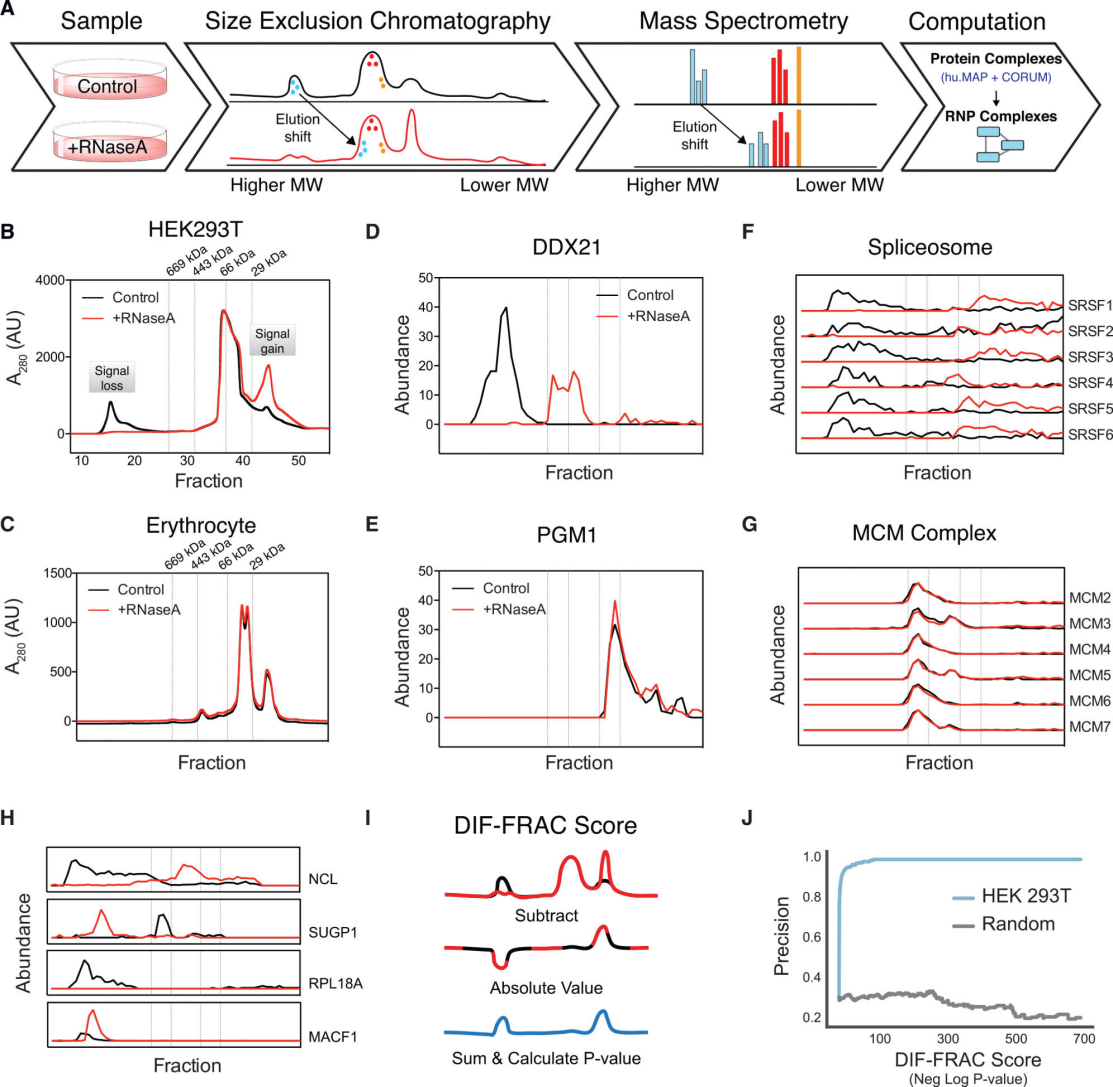
Differential Fractionation (DIF-FRAC) Identifies RNP Complexes (A) The DIF-FRAC workflow requires two equivalent cell culture lysates for a control and an RNase A-treated sample. Lysate is separated into fractions using size-exclusion chromatography (SEC), and proteins in each fraction are identified using mass spectrometry to determine individual protein elution profiles proteome-wide for each condition. An elution shift of a protein upon RNase A treatment is indicative of an RNA-protein association. Elution shifts are cross-referenced with known protein complexes to identify RNP complexes. (B) Separations of HEK293T lysate under control (black) and RNase A-treated (red) conditions monitored by bulk SEC absorbance profiles at A_280_ show loss of high-molecular weight signal upon treatment. (C) Negative control separations of erythrocyte lysate under control (black) and RNase A-treated (red) conditions monitored by bulk SEC absorbance profiles at A_280_ show no change in absorbance signal. (D) RNA-binding protein elution profile for positive control nucleolar RNA helicase 2 (DDX21) (abundance = count of unique PSMs). The elution profile shows sensitivity to RNase A treatment. (E) Elution profile for negative control phosphoglucomutase (PGM1) is not sensitive to RNase A treatment. (F) Elution profiles for subunits of the spliceosome RNP complex (i.e., positive control) show co-elution of complex in control and a shift in elution upon RNase A treatment. (G) Elution profile for the non-RNA-associated MCM complex (i.e., negative control) shows no detectable elution shift. (H) Example traces of four known RNA-binding proteins exhibiting different behaviors of elution profile changes upon RNase A treatment. NCL shows a loss in molecular weight, while SUGP1 shows an increase in molecular weight. RPL18A shows a decrease in observed abundance, while MACF1 shows an increase in observed abundance. In (B)–(H), dashed lines correspond to the elution volumes of molecular weight standards thyroglobulin (M_r_ = 669 kDa), apoferritin (M_r_ = 443 kDa), albumin (M_r_ = 66 kDa), and carbonic anhydrase (M_r_ = 29 kDa). Molecular weight labels on subsequent plots are removed for clarity. (I) A DIF-FRAC score is calculated for each protein from the absolute value of the difference of the elution profiles between control and RNase A-treated samples, and then summed. A p value is then calculated from a *Z* score compared to a background distribution of DIF-FRAC scores preserving the rankings among proteins. See also Figure S2A. (J) DIF-FRAC p value calculated on HEK293T data shows strong ability to discriminate known RNA-binding proteins from other proteins. See also Figure S2B.

Here, we present differential fractionation (DIF-FRAC) for interaction analysis, which measures the sensitivity of protein complexes to a given treatment (e.g., RNase A) using native size-exclusion chromatography followed by mass spectrometry. DIF-FRAC is based on a high-throughput co-fractionation mass spectrometry (CF-MS) approach that we developed and applied to a diverse set of tissues and cells types en route to generating human and metazoan protein complex maps (Drew et al., 2017a; Havugimana et al., 2012; Wan et al., 2015). DIF-FRAC builds upon CF-MS by comparing chromatographic separations of cellular lysate under control and RNA-degrading conditions (Figure 1A). A statistical framework is then applied to discover RNP complexes by identifying concurrent shifts of known protein complex subunits upon RNA degradation (Figure 1A).

Analysis of DIF-FRAC data answers important questions as to the role of RNA plays in macromolecular complexes. Specifically, we identify RNP complexes that (1) dissociate, (2) form stable protein-only complexes also in the absence of RNA, and (3) change composition in the absence of RNA, suggesting specific roles for RNA in each of these cases. The technical flexibility of DIF-FRAC in discovering RNA-associated interactions can potentially be expanded to virtually any tissue and organism. To demonstrate the versatility of our method to additional non-human samples, we apply DIF-FRAC to mouse embryonic stem cells (mESCs), identifying 1,165 RNA-associated proteins, to show that the method is highly adaptable and can be extended to discover RNP complexes in diverse samples.

Finally, we created a system-wide resource of 1,428 RNP complexes, many of which are previously unreported as having an RNA component, representing 20% of known human protein complexes. We provide our resource, rna.MAP, to the community as a fully searchable web database at rna.proteincomplexes.org.

## RESULTS AND DISCUSSION

### Differential Fractionation (DIF-FRAC) Identifies RNP Complexes

The DIF-FRAC strategy builds upon our previous strategy of CF-MS for identifying protein complexes in cellular lysate (Havugimana et al., 2012; Wan et al., 2015). CF-MS chromatographically separates protein complexes into fractions and uses a mass spectrometry pipeline to identify resident proteins in each fraction. The chromatographic elution profile of each protein is correlated to elution profiles from other proteins, and similar profiles suggest physical interactions. Likewise, the DIF-FRAC strategy detects RNP complexes by identifying changes in the CF-MS elution profile of a protein complex’s subunits upon degradation of RNA (Figure 1).

We applied DIF-FRAC to human HEK293T cell lysate using size-exclusion chromatography (SEC) to separate the cellular proteins in a control and an RNase A-treated sample into 50 fractions (Figure 1A). During cell lysis and chromatographic separation, lysate is diluted approximately 1,000-fold, which mitigates spurious association of molecules (see STAR Methods). Upon RNase A treatment, we observed a loss in the bulk A280 chromatography absorbance signal in the high molecular-weight regions and an increase in absorbance in lower molecular weight regions. Protein and to a lesser degree nucleic acid show absorbance at A280, and therefore this is consistent with higher molecular weight RNP species (>1,000 kDa) becoming lower molecular weight species in the absence of RNA (Figure 1B). The distribution of cellular RNA in these fractions measured using RNA sequencing (RNA-seq) confirmed that we are accessing a diverse RNA landscape of mRNAs, small RNAs, and long non-coding RNAs (lncRNAs) (Figure S1). As a negative control, we applied the same DIF-FRAC strategy to human erythrocytes, which we reasoned should have fewer RNPs because they have substantially lower amounts of RNA because of the loss of their nucleus and ribosomes upon maturation (Keerthivasan et al., 2011; Lee et al., 2014). Accordingly, the absorbance chromatography signal of erythrocyte lysate showed only a negligible difference in a DIF-FRAC experiment (Figures 1C and S1B). Together, these data establish that DIF-FRAC is capable of identifying bulk changes to the RNA-bound proteome.

We next used mass spectrometry to identify and quantify the resident proteins in each fraction for both the control and RNase A-treated chromatographic separations, resulting in 8,376 protein identifications (unique peptide spectral matches ≥ 2). Using these abundance measurements, we compared elution profiles (i.e., abundance change across chromatographically separated molecular weights) between the control and RNase A-treated experiments for each protein. A shift in a protein’s elution profile between experiments is indicative of a protein-RNA interaction. For example, the known RNA helicase DDX21 shows a substantial shift in its elution profile upon RNase A treatment (Figure 1D), consistent with DDX21’s known association with RNA (Calo et al., 2015). Alternatively, proteins such as the glucose synthesis enzyme, PGM1, show no shift, consistent with its not binding RNA (Figure 1E).

We can further examine these elution profile differences in the context of physically associated proteins to identify RNP complexes. For example, subunits of the spliceosome, a known RNP complex, show elution profiles that co-elute in the control but shift markedly upon RNA degradation (Figure 1F). In contrast, the elution profiles of subunits of the non-RNA-associated hexameric MCM complex (M_r_ ~ 550 kDa) (Figure 1G), as well as the eight-subunit COP9 signalosome (M_r_ ~ 500 kDa; Oron et al., 2002) (Figures S1C and S1D), are unchanged by RNase A treatment, consistent with the complexes’ not interacting with RNA. Thus, DIF-FRAC produces a robust signal that can be used to differentiate between non-RNA-associated complexes and RNP complexes.

### Systematic Identification of RNP Complexes

In order to systematically identify RNP complexes in a DIF-FRAC experiment, we first developed a computational framework to identify statistically significant changes in individual proteins’ elution behavior to identify RNA-associated proteins. We observed a variety of changes in elution behavior of known RNA-binding proteins upon RNase A treatment, including decrease in molecular weight (e.g., NCL), increase in molecular weight (e.g., SUGP1), decrease in observed abundance (e.g., RPL18A), and increase in observed abundance (e.g., MACF1) (Figure 1H). To capture this range of behaviors in a simple metric, we developed the “DIF-FRAC score,” which evaluates the degree to which two chromatographic separations differ (Figure 1I). Briefly, the DIF-FRAC score is a normalized Manhattan distance between a protein’s control and RNase A-treated elution profiles (see STAR Methods). To identify significant changes, we calculated p values by comparing each protein’s DIF-FRAC score with an abundance-controlled background distribution of DIF-FRAC scores from non-RNA-associated proteins (Figure S2A; see STAR Methods for full description). We evaluated the score’s performance on a curated set of known RNA-associated proteins and saw strong correspondence between precision and high-ranking proteins (Figure 1J; Figure S2B). Figure 1J specifically illustrates that for high-scoring proteins nearly all have been previously identified as RNA binding, demonstrating the accuracy of our computational method, which does not use any prior functional or literature information to prioritize previously identified RNA-binding proteins. In replicate experiments, we see similar performance (Figures S2E and S2F) as well as high correlation of the DIF-FRAC score among replicates (Figure S2G). Furthermore, replicates show high levels of correlation among elution profiles in both control (Figure S2H) and RNase A (Figure S2I) experiments. We additionally observe high levels of correlation between adjacent fractions in experiments, suggesting that protein identifications and subsequent DIF-FRAC score calculations are well supported and robust (Figure S2J). Finally, we see the expected shift in total peptide spectral matches (PSMs) (Figure S2K) as well as total proteins identified (Figure S2L) from high-molecular weight fractions to low molecular weight fractions as the majority of RNA-associated proteins will lose molecular weight upon RNase A treatment.

DIF-FRAC identifies 1,012 proteins with significant elution profile differences in HEK293T cells with a false discovery rate (FDR) cutoff of 5% (Table S1). To validate our metric, our set of statistically significant hits was compared with RNA-binding proteins identified from 11 other studies using alternative methods, including RNA interactome capture (RIC) (Baltz et al., 2012; Castello et al., 2012), organic phase separation (Queiroz et al., 2019; Trendel et al., 2019), and others (Bao et al., 2018; Beckmann et al., 2015; Castello et al., 2016; Conrad et al., 2016; Hentze et al., 2018; Huang et al., 2018; Kramer et al., 2014). These results indicate that the DIF-FRAC score is highly accurate for identifying individual RNA-associated proteins (Figures S3A–S3K). We also compared with two other methods, an indirect method (Brannan et al., 2016) (Figure S3L) and another that uses a similar strategy of RNase treatment followed by density gradient ultracentrifugation (Caudron-Herger et al., 2019) (Figure S3M), and saw similar performance.

**Figure 2.**
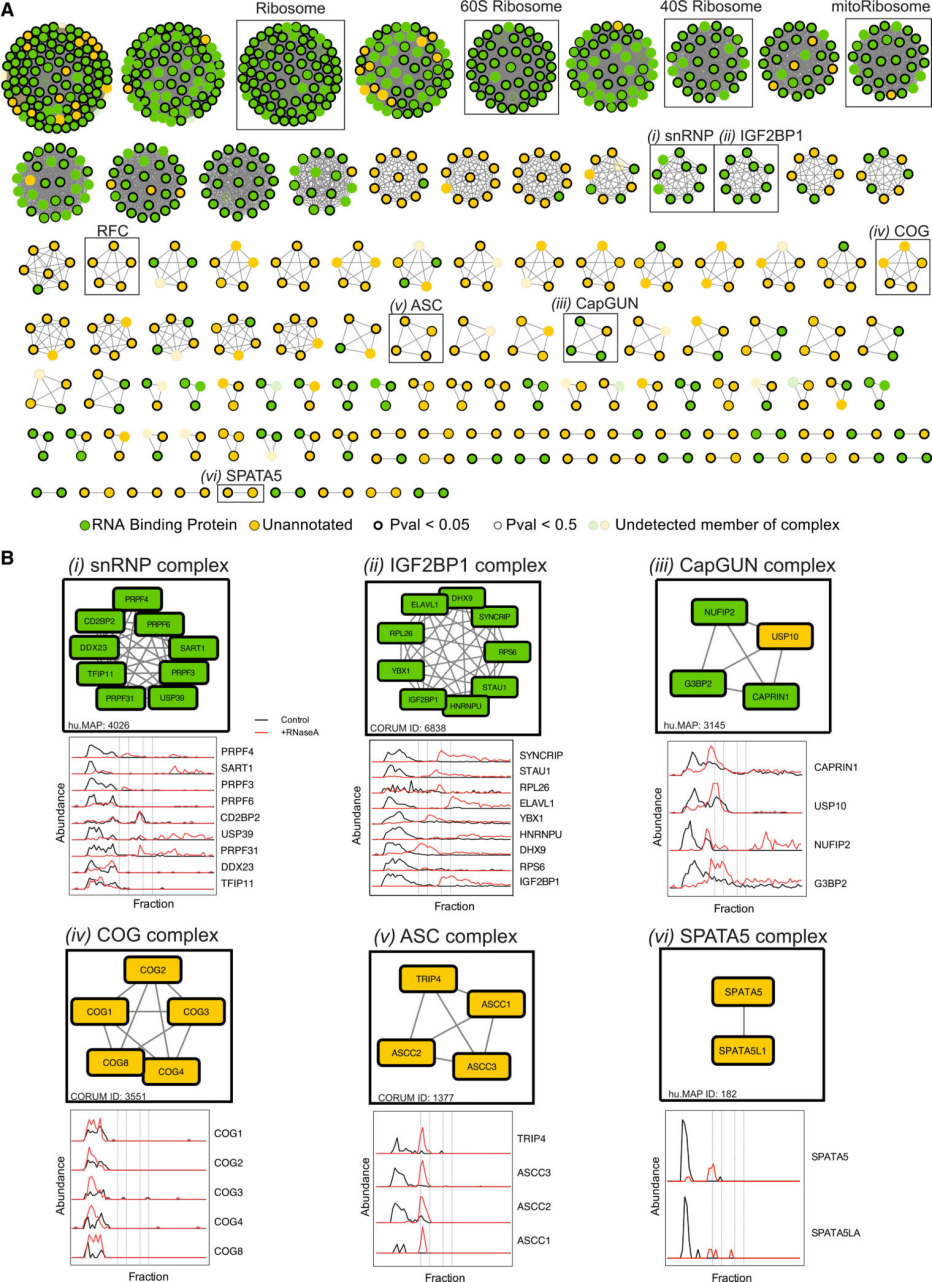
DIF-FRAC Reveals a Map of Stable RNP Complexes (A) One hundred fifteen RNP complexes identified by the DIF-FRAC method termed “RNP Select.” Green nodes represent RNA-binding proteins annotated as “RNP complex” in UniProt, and yellow nodes are unannotated proteins. Nodes with thick black border and thin black border represent p values < 0.05 and < 0.5, respectively. Transparent nodes represent undetected members of the complex in our proteomic experiments. RNP Select complexes are defined as complexes whose protein subunits co-elute in the control DIF-FRAC sample (>0.75 average correlation coefficient), and >50% of subunits have DIF-FRAC p values > 0.5. DIF-FRAC identified many known RNP complexes, such as the ribosome, mitochondrial ribosome, and snRNP, as well as novel RNP complexes such as RFC, COG, ASC, and SPATA5. (B) Individual RNP complexes with elution profiles, including (i) snRNP, (ii) IGF2BP1, (iii) CapGUN, (iv) COG, (v) ASC, and (vi) SPATA5. Abundance represents count of unique PSMs for each protein. See also Figure S4.

To expand on previous systematic studies of RNA-binding proteins, we exploited the unique features of DIF-FRAC to identify which RNA-associated proteins are assembled into higher order RNP complexes. Specifically, we searched for protein complexes whose subunits co-elute in the control experiment in addition to being sensitive to RNase A treatment (e.g., see Figure 1F). We detected 115 RNP complexes that fit these criteria, which we term “RNP Select” (Figure 2; Table S2). The RNP Select set consists of 464 unique proteins, and importantly, it recapitulates many known RNP complexes. The set includes canonical RNPs such as the 40S ribosome and the spliceosomal tri-snRNP complex, a major component of the catalytically active spliceosome that contains an intricate network of snRNA binding interactions (Agafonov et al., 2016) (Figure 2B). The set also includes RNPs with more specific functions, such as the IGF2BP1 complex, which is involved in RNA stability (Weidensdorfer et al., 2009) (Figure 2B). Because these data demonstrated the veracity of the DIF-FRAC strategy, we next searched our dataset for additional insights into RNP biology.

First, the RNP Select set provided new details about known RNPs. For example, stress granules are large membrane-less organelles that sequester mRNAs and prevent translation (Lin et al., 2015; Mittag and Parker, 2018; Spector and Lamond, 2011) and contain RNA-associated proteins including CAPRIN1, G3BP2, USP10, and NUFIP2, each localizing to stress granules (Bardoni et al., 2003; Matsuki et al., 2013; Solomon et al., 2007). Interestingly, our previous map of human protein complexes (Drew et al., 2017a) suggests that the known complex of G3BP, CAPRIN, and USP10 (Kedersha et al., 2016) also physically interacts with NUFIP2, leading us to suggest the name CapGUN (i.e., CAPRIN1, G3BP2, USP10, NUFIP2). Importantly, DIF-FRAC revealed that CapGUN subunits co-elute and associate with RNA (Figure 2B).

More important, RNP Select also contains several complexes not previously known to associate with RNA. For example, the spinal muscular atrophy associated activating signal cointegrator(ASC) complex (Knierim et al., 2016) (Figure 2B) is a transcriptional coactivator of nuclear receptors and has a role in transactivation of serum response factor (SRF), activating protein 1 (AP-1), and nuclear factor kappaB (NF-kappaB) (Jung et al., 2002). Upon RNase A treatment, we observed a substantial shift in elution from a high molecular weight to a lower molecular weight for all subunits of the ASC complex, strongly suggesting that the complex associates with RNA (Figure 2). Interestingly, one ASC component, ASCC1, has a predicted RNA-binding motif near its C terminus and has been shown to localize to nuclear speckles (Soll et al., 2018), which like stress granules are membrane-less organelles enriched for RNPs. Our results, in coherence with previous studies, point to a role for the ASC complex associating with RNA in RNP granules. Other notable examples of previously uncharacterized RNP complexes include the conserved oligomeric Golgi (COG) complex, which is involved in intra-Golgi trafficking, and the SPATA5-SPATA5L1 complex, an uncharacterized complex linked to epilepsy, hearing loss, and mental retardation syndrome (Tanaka et al., 2015) (Figure 2B), among others (Table 1).

The DIF-FRAC method identifies RNP complexes using biochemical separation of cellular lysate. To demonstrate that RNP complexes identified by DIF-FRAC behave in a coordinated fashion *in vivo*, we looked in enhanced cross-linking and immunoprecipitation (eCLIP) data from the ENCODE project (Van Nostrand et al., 2016) to determine whether co-complex proteins bound RNA in a similar fashion. Figure S4A shows elution profiles for U4/U6-U5 tri-snRNP complex subunits PRPF4, PRPF8 and EFTUD2 co-elute in the control experiment and change their elution profile upon RNase A treatment. Figure S4B shows sequencing reads of the RRBP1 mRNA from eCLIP experiments of the same subunits. All three subunits show a similar pattern of binding the RRBP1 mRNA, suggesting that they are binding as a complex *in vivo*. This provides evidence of co-complex proteins identified as an RNP by DIF-FRAC behaving in a coordinated fashion *in vivo*.

Finally, to ascertain the total number of annotated protein complexes that likely function with an RNA component, we evaluated DIF-FRAC evidence for RNA-associated proteins in addition to the 11 other studies targeting direct RNA-binding proteins (described above) and identify 1,428 complexes that contain a majority of RNA-associated proteins (see STAR Methods). This analysis suggests that greater than 20% of known protein complexes associate with RNA (Table 1; Table S2). We provide the complete set of RNP complexes as a fully searchable web database, rna.MAP, at http://rna.proteincomplexes.org. This represents a detailed resource of human RNP complexes, providing myriad testable hypotheses to guide further explorations of RNP biology.

### Validation of RNP Complexes Using RNA Hairpin Pull-Down Experiments

To validate RNP complexes identified by our DIF-FRAC method, we reanalyzed an orthogonal proteomics dataset on the basis of a pull-down approach using microRNA (miRNA) hairpins as bait. Treiber et al. (2017) immobilized 72 different pre-miRNAs on beads and incubated with lysate from 11 different cell lines, resulting in more than 3,000 proteomic experiments. Although the RNA probes used were originally from pre-miRNAs, the number of probes provides a large sample in which to query protein-RNA interactions. The pull-down nature of these experiments keeps protein complexes intact when binding RNA, which allowed us to reinterpret the data in order to independently ascertain each complex’s ability to associate with RNA. We first reprocessed all pull-down experiments using our protein identification pipeline (Table S4) and then asked if our set of RNP complexes were identified. As a background to compare against, we used the 4,429 complexes in CORUM and hu.MAP, which were not identified as RNP complexes. Figure S4C shows both RNP complexes and RNP Select complexes are identified and more abundant on average than non-RNP complexes in RNA hairpin pull-down experiments (p = 0.0 and 5.18e-44, respectively, Mann-Whitney test). Additionally, Figure S4D shows that RNP complexes identified only in this study are also identified and more abundant on average than non-RNP complexes (p = 3.33e-08, Mann-Whitney test). We next looked at specific examples of RNP complexes within the RNA hairpin pull-down experiments (Figures S4E–S4H). In particular, we observed that the novel RNP NIPSNAP1/2 complex, the prohibitin-2 complex and the SPATA complex were all identified in a select subset of pull-down experiments. The Microprocessor complex in Figure S4E serves as a positive control. These data provide independent confirmation of the novel DIF-FRAC RNP complexes’ association with RNA.

### Classification of RNP Complexes

**Figure 3.**
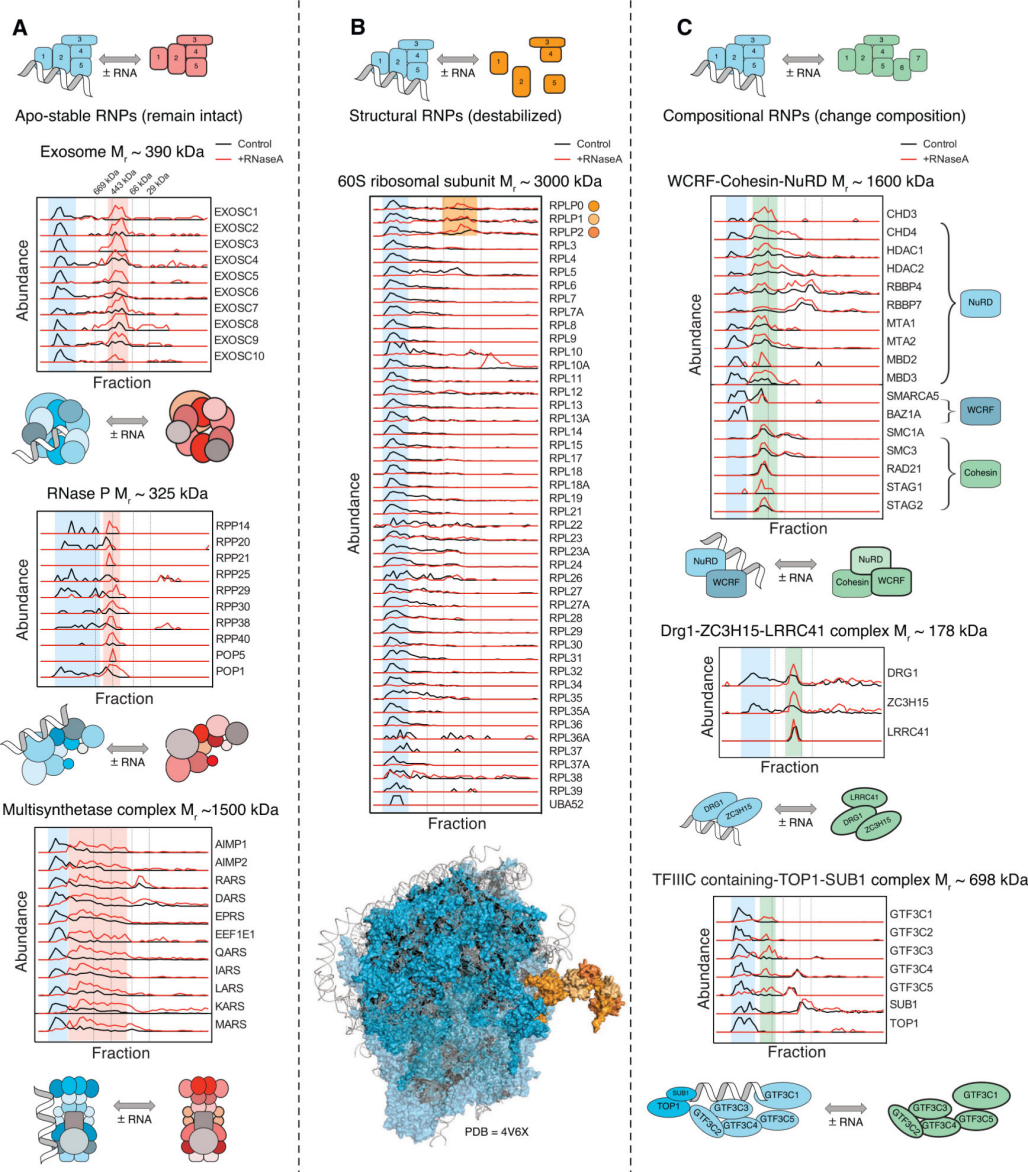
DIF-FRAC Identifies Three Classes of RNP Complexes (A) “Apo-stable” RNP complexes: elution profiles of the exosome (top, CORUM: 7443), RNase P (middle, CORUM: 123), and the multi-synthetase complex (bottom, CORUM: 3040) show that each complex is a stable complex that binds RNA, and the complex remains intact in the absence of RNA. Blue shading represents RNA-bound form, and red shading represents RNA-unbound complex. See also Figure S5. (B) “Structural” RNP complexes: elution profiles of the 60S ribosomal subunit (CORUM: 308) show that the complex destabilizes upon RNA degradation, and subunits no longer co-elute upon RNase A treatment. DIF-FRAC elution data show the ribosomal subunits RPLP0, RPLP1, and RPLP2 (orange) remain as a subcomplex upon RNA degradation, consistent with their position in the solved ribosome structure whose interactions are not mediated by RNA(bottom, PDB: 4V6X, protein in blue, RNA in gray, ribosomal stalk in orange). (C) “Compositional” RNP complexes. Top: elution profiles of WCRF-Cohesin-NuRD (CORUM: 282) and NuRD-WCRF suggest that RNA association promotes different forms of the complex. Middle: elution profiles of Drg1-ZC3H15-LRRC41 complex (hu.MAP: 2767), which forms only in the absence of RNA. Bottom: elution profiles of the TFIIIC-containing TOP1-SUB1 complex (CORUM: 1106) loses two subunits, TOP1 and SUB1, upon RNA degradation. Green shading represents RNA-unbound complex. Vertical dashed lines correspond molecular weight standards described in Figure 1. See also Figure S5.

RNA performs a variety of roles in macromolecular complexes. For example, it can bind as a substrate, function as an integral structural component, or act as a regulator of a complex’s composition. Mirroring these roles, DIF-FRAC data reveal that upon RNA degradation, the proteins in RNP complexes can remain in an intact complex (Figure 3A), become destabilized (Figure 3B), or adopt different higher order configurations (Figure 3C). We therefore categorize RNP complexes into three groups.

The first category, which we term “apo-stable,” defines protein complexes that remain stable after RNase A treatment. These include the exosome, RNase P, and the multi-synthetase complex (Figure 3A). Elution profiles of apo-stable complexes show that in the absence of RNA, subunits still co-elute but do so as a lower molecular weight complex. Available atomic structures of the exosome complex with (Figure S5A) and without RNA (Figure S5B) support the concept that RNA is peripheral to the stability of the complex (Gerlach et al., 2018; Liu et al., 2006; Weick et al., 2018).

The second category, which we designate as “structural,” refers to complexes for which RNA is essential for the RNP complex structure and/or subunit solubility. These include, for example, the 60S and 40S ribosomal subcomplexes (Figure 3B; Figure S5C). Upon degradation of RNA, the observed abundance of ribosomal protein subunits markedly decreases, suggesting that the ribosome breaks apart and subunits become insoluble. This result is consistent with solved structures of the ribosome (Anger et al., 2013), demonstrating the centrality of rRNAs to the overall complex architecture (Figure 3B). Interesting exceptions to this behavior are the DIF-FRAC elution profiles for RPLP0, RPLP1, and RPLP2. These proteins co-elute in the RNase A-treated sample, suggesting RNA does not mediate their interaction. Strikingly, however, this observation is consistent with the atomic structure of the human ribosome, which suggests that interactions between RPLP0, RPLP1, and RPLP2 are entirely protein mediated (Figure 3B). This example demonstrates how DIF-FRAC data can not only identify RNA-protein-mediated interactions but can also provide structural information about RNP subcomplexes, similar to how we have shown previously that CF-MS experiments provide structural information (Drew et al., 2017b; Wan et al., 2015). Another subunit that has peculiar behavior is the RPS3 subunit in the 40S subcomplex (Figure S5C), which still elutes in high-molecular weight fractions after RNase A treatment. Interestingly, RPS3 is known to have an extraribosomal role in DNA damage response (Kim et al., 1995) and RPS3’s interaction with non-ribosomal proteins is likely why it behaves differently than other ribosomal subunits. This example demonstrates how DIF-FRAC data can be used to identify potential moonlighting functions for individual subunits.

The third category, “compositional” complexes, refers to those in which RNA promotes different stable combinations of protein-complex subunits, perhaps in a regulatory role (Figure 3C). For example, the WCRF (Williams syndrome transcription factor-related chromatin remodeling factor) complex, NuRD (nucleosome remodeling deacetylase) complex, and Cohesin complex are reported to assemble into a chromatin-remodeling supercomplex (CORUM: 282). We observed the WCRF and NuRD complexes co-eluting in the control experiment, forming a 12-subunit complex that shifts its elution upon RNA degradation. Interestingly, we also observed the supercomplex (WCRF, NuRD, and Cohesin) eluting as a ~17-subunit complex in the RNA degradation condition. This composition change provides an explanation for why several NuRD-containing complexes are observed experimentally (Hakimi et al., 2002; Xue et al., 1998); our data suggest that these may represent both RNP complexes and non-RNA-associated complexes.

We also identified an uncharacterized compositional RNP complex containing the cell growth regulators DRG1 and ZC3H15 (DRFP1) (Ishikawa et al., 2005) that are implicated in lung cancer (Lu et al., 2016). ZC3H15 stabilizes DRG1 and prevents degradation possibly by preventing poly-ubiquitination (Ishikawa et al., 2005). Our result suggests that RNA is also involved in ZC3H15’s role in stabilizing DRG1, as we observed a shift to a non-RNA-associated complex containing DRG1-ZC3H15 and LRRC41 in the absence of RNA (Figure 3C). LRRC41 is a probable substrate recognition component of E3 ubiquitin ligase complex (Kamura et al., 2004).

A further example of a compositional RNP complex is the transcription factor (TF) IIIC-TOP1-SUB1 complex, which is involved in RNA polymerase III pre-initiation complex (PIC) assembly (Male et al., 2015). DIF-FRAC shows that this seven-subunit complex changes composition to the five-subunit TFIIIC upon RNA degradation (Figure 3C), offering further insights into the mechanism of TFIIIC-dependent PIC formation.

Finally, we identified the chromatin remodeling BRG/hBRM-associated factors (BAF; the mammalian SWI/SNF complex; SWI/SNF-A) and polybromo-associated BAF (PBAF; SWI/SNF-B) complexes as compositional RNP complexes, which is significant because these are some of the most frequently mutated protein complexes in cancer (Hodges et al., 2016; Tang et al., 2017) (Figures S5D–S5G). BAF and PBAF complexes share a set of common core subunits, but also each has signature subunits that are related to their respective functions. Elution profiles in both replicates show these core subunits co-elute with PBAF-only subunits in the control but co-elute with BAF-only subunits upon RNA degradation (Figures S5D and S5E). An exception to this is the ARID2 subunit, which clusters between BAF and PBAF-only subunits (Figure S5G). These data suggest BAF exists as a non-RNA-associated complex, while PBAF functions as an RNP complex (Figure S5F), consistent with its known role in transcription and supporting a previously described RNA-binding model whereby lncRNAs interact with SWI/SNF complexes in cancer (Tang et al., 2017). Together, these examples demonstrate the power of DIF-FRAC to describe the various physical relationships between RNA and macromolecular protein complexes.

### Characterization of Individual RNA-Associated Proteins

Although our efforts focused primarily on higher order RNP complexes, it is important to note that DIF-FRAC is also a powerful complement to existing methods for characterizing individual RNA-associated proteins. Indeed, DIF-FRAC identified 196 human RNA-associated proteins not previously identified in the many previous studies discussed in the Introduction (Table S3; Figure S3N). These DIF-FRAC identified RNA-associated proteins were strongly enriched in RNA-binding domains annotated by Interpro (Finn et al., 2017) (Figure S3O). To further validate these novel RNA-associated proteins, we compared their propensity to be pulled down by RNA hairpins from Treiber et al. (2017) to a random set of proteins. In Figure S6A, we see enrichment of RNA hairpin pull-down experiments that identify novel DIF-FRAC proteins over randomly selected proteins. We also see an increase in the percentage of RNA binding annotated co-complex interactors in the novel proteins compared with randomly selected proteins (p = 1.3e-04, Mann-Whitney test) (Figure S6B). These results strongly validate the novel RNA-associated proteins and provide additional overall confidence to the DIF-FRAC method’s ability to identify RNA-associated proteins.

**Figure 4.**
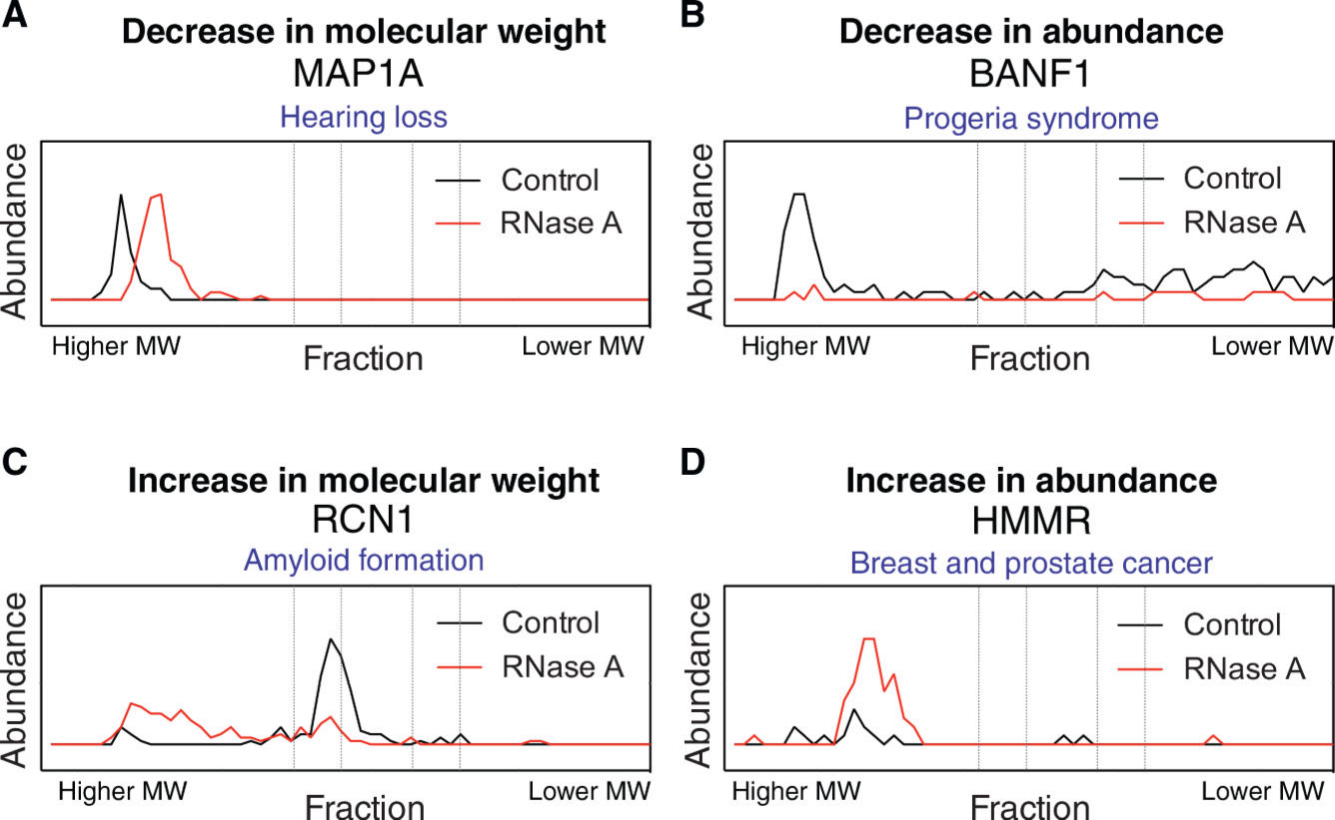
DIF-FRAC Identifies Four Distinct Signals for RNA-Associated Proteins (A–D) Examples of elution profiles for disease related proteins that (A) decrease in size, MAP1A; (B) decrease in observed abundance (less soluble), BANF1; (C) increase in size, RCN1; and (D) increase in observed abundance (more soluble), HMMR, upon RNA degradation. See also Table S3 and Figure S6.

As we described above, inspection of elution profiles for the individual proteins revealed at least four distinct DIF-FRAC signals (Figures 1H and 4). These manifest as elution-profile shifts with RNase A treatment that show (1) an apparent decrease in molecular weight of the RNA-associated protein consistent with the degradation of an RNA component (Figure 4A); (2) a decrease in observed abundance, suggesting the RNA-associated protein becomes insoluble or is degraded (Figure 4B); (3) an apparent increase in molecular weight, suggesting the RNA-associated protein forms a higher order species or aggregate (Figure 4C); or (4) an increase in observed abundance, indicative of the RNA-associated protein becoming more soluble (Figure 4D).

Analysis of all identified RNA-associated proteins shows 796 (79%) decrease in molecular weight, while 216 RNA-associated proteins (21%) increase in size (Figure S6C). Aside from RNA acting as an interaction partner to RNA-associated proteins, RNA has been shown to regulate the oligomerization state of proteins both positively (Bleichert and Baserga, 2010; Huthoff et al., 2009; Xie et al., 2018) and negatively (Yoshida et al., 2004). Our data suggest that although the majority of RNA-associated proteins form higher order assemblies with RNA, the oligomerization of 21% is potentially inhibited by RNA. Alternatively, RNA has also been shown to alter the solubility state of proteins (Maharana et al., 2018). We observe an increase in observed abundance for 535 proteins (53%) upon RNase A treatment, a decrease in abundance for 470 proteins (47%), and no change in observed abundance for only 7 proteins. This suggests RNA affects the solubility for most RNA-associated proteins and may function to tune protein availability in the cell.

Looking specifically at individual proteins provided insights that could affect our understanding of human disease. For example, we found that BANF1, a chromatin organizer, appears insoluble under our experimental conditions without RNA (Figure 4B). Interestingly, the BANF1 mutation Ala12-Thr12 causes Hutchinson-Gilford progeria syndrome, a severe and debilitating aging disease, by a reduction in protein levels (Puente et al., 2011). Our data suggest the hypothesis that this reduction is caused by disruption of the RNA-BANF1 interaction, leading to insolubility and degradation. Furthermore, RNA has also been shown to solubilize proteins linked to pathological aggregates (Maharana et al., 2018). Our data identify a number of CREC family members (CALU, RCN1, RCN2, and SDF4; Figure 4C; Table S1) as RNA-associated proteins that increase in molecular weight upon RNA degradation. The CREC family is a group of multiple EF-hand, low-affinity calcium-binding proteins with links to amyloidosis (Vorum et al., 2000). DIF-FRAC demonstrates a dependence of RNA on the oligomerization state of CALU, which could play a role in the formation of amyloid deposits similar to that observed for prion-like RNA-associated proteins (Maharana et al., 2018). On the basis of these examples and the many disease links to DIF-FRAC identified RNP complexes (Table 1), we anticipate that our data will generate testable RNA-related hypotheses about disease-related states.

### Directed Validation of Replication Factor C (RFC) as an RNP Complex

**Figure 5.**
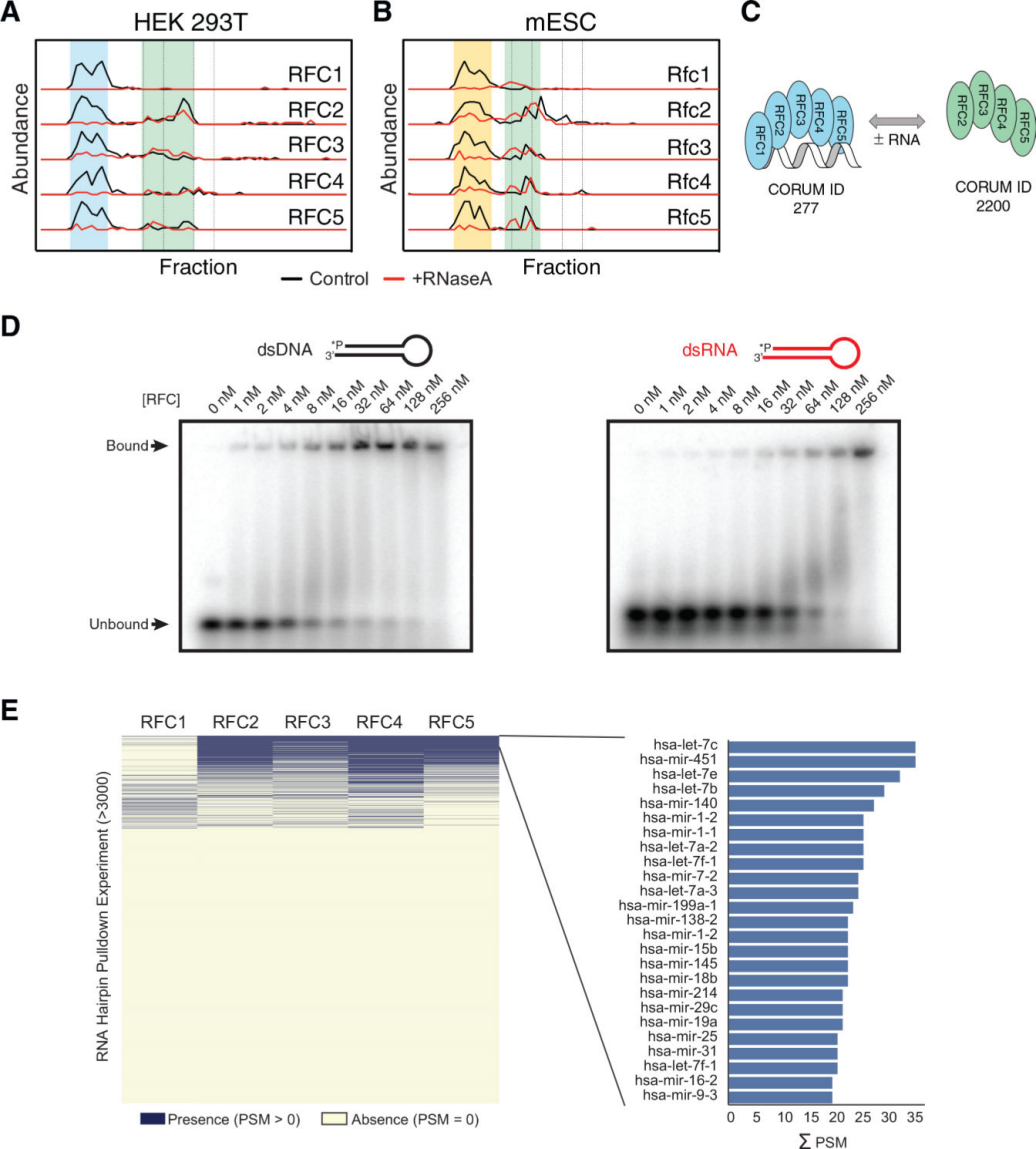
RFC Is an RNP Complex (A and B) Elution profiles in both human (A) and mouse (B) demonstrate that RFC1–5 forms an RNP complex (blue/yellow highlight). A smaller subcomplex of RFC2–5 (green highlight) becomes the dominant form upon RNA degradation. (C) A cartoon to show the RNA dependence of annotated complexes RFC1–5 (blue) and RFC2–5 (green) as determined by DIF-FRAC. RNA is shown in gray. (D) Electromorphic mobility shift assays (EMSA) of various concentrations of purified S. *cerevisiae* RFC mixed with 1 nM ^32^P-labeled oligonucleotides. Representative gels show that RFC binds dsDNA and dsRNA substrates. RFC-nucleic acid complexes were separated on 10% native gels. Binding constants are in the nanomolar range (see also Figure S7). (E) RFC component identification in RNA hairpin pull-down experiments (right panel) and the top 25 hairpin pull-downs on the basis of the sum of PSMs (left panel).

An important aspect of DIF-FRAC is that although it provides a systematic survey, the experimental basis for each data point can be directly assessed in the elution profiles. Nonetheless, the ultimate demonstration of the utility of any large-scale dataset is its ability to make predictions that can be validated by orthogonal experiments. Among the most surprising findings in our data was that the extensively characterized RFC complex (Yao and O’Donnell, 2012) exists as a stable RNP complex (Figure 2A). During replication and DNA damage repair, the RFC complex is responsible for loading PCNA, a DNA polymerase processing factor, onto DNA. Although previous RNA binding studies have identified individual subunits as interacting with RNA (Bao et al., 2018; Trendel et al., 2019), the RFC complex has not been previously described as an RNP. Strikingly, DIF-FRAC identified two previously characterized variants of the RFC complex, RFC1–5 and RFC2–5 (Figure 5A), and more important demonstrated that RFC1–5 appears to be the dominant variant and is also the RNA-associated form (Figure 5C). Consistent with the RFC complex interacting with RNA, the homologous clamp loader in *E.coli*, γ complex, is known to load the DNA clamp onto RNA-primed template DNA (Yao and O’Donnell, 2012), and eukaryotic RFC has also been shown to be capable of loading PCNA onto synthetic RNA-primed DNA (Yuzhakov et al., 1999). In light of this finding, we tested whether purified RFC complex from *S. cerevisiae* could directly bind different species of nucleic acids. We observed that RFC not only binds double-stranded DNA (dsDNA) but also binds double-stranded RNA (dsRNA) with surprisingly tight binding constants in the nanomolar range (Figure 5D; Figure S7).

To further validate the RFC complex as an RNP, we searched for RFC subunits in the reanalyzed RNA hairpin pull-down experiments. Figure 5E shows RFC subunits are pulled down by RNA and are relatively promiscuous binders but are not general RNA hairpin binders, as they are identified in only ~10% of experiments. More important, we observed the majority of RFC components to be identified in the same set of experiments, strongly suggesting the RFC subunits interact with RNA as an assembled complex. These data thus show that RFC binds dsRNA and point to an uncharacterized role for RNA in the function of RFC. In addition these results further validate the use of DIF-FRAC to identify uncharacterized RNP complexes.

### Evaluating RNPs in Multiple Proteomes

**Figure 6.**
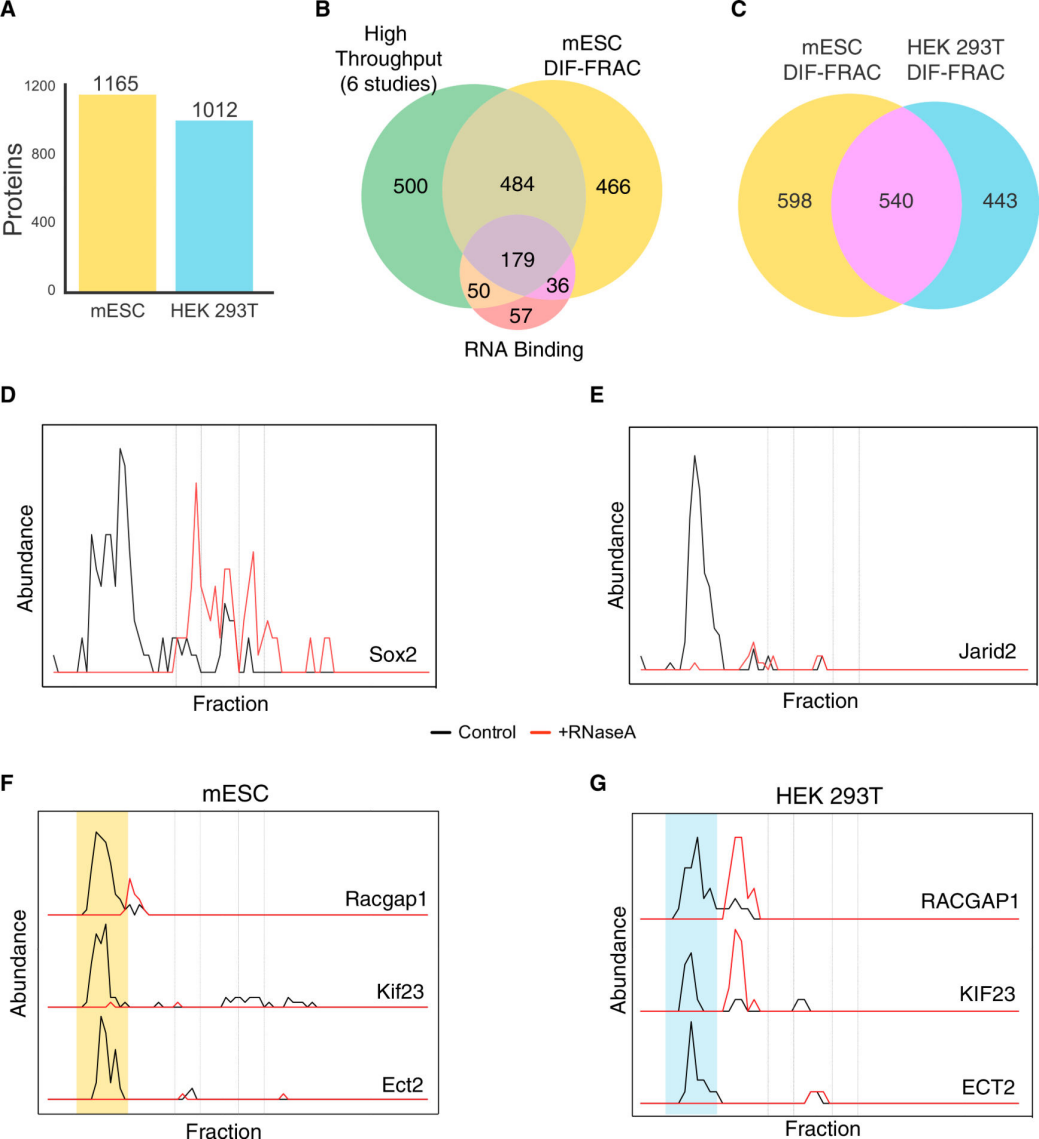
DIF-FRAC Identifies RNP Complexes across Cell Types and Species (A) DIF-FRAC identifies 1,165 RNA-associated proteins in mESCs (mouse embryonic stem cells) and 1,012 RNA-associated proteins in HEK293T cells. (B) Venn diagram of considerable overlap between previously published large-scale RNA-protein interaction studies, literature-annotated RNA-binding proteins, and DIF-FRAC-identified RNA-associated proteins in mESCs. (C) RNA-associated human-mouse orthologs are identified reproducibly in DIF-FRAC experiments. (D and E) Elution profiles for known pluripotency factors Sox2 (D) and Jarid2 (E) show association with RNA in mESCs. (F and G) Elution profiles of the centralspindlin complex for (F) mESCs and (G) HEK293T cells demonstrate that centralspindlin is an RNP complex in both species. Yellow and blue shading represents RNA-bound complex in mESCs and HEK293T cells, respectively.

Finally, because DIF-FRAC does not rely on any specialized reagents, the strategy can be applied to any cell type that can be readily isolated. Because of the long-standing interest in the role of RNPs in embryonic development (e.g., for targeted localization of maternal RNAs [Escobar-Aguirre et al., 2017], processing of non-coding RNA to direct differentiation and stem cell potency [Dinger et al., 2008; Guttman et al., 2011; Yan et al., 2013]), we applied DIF-FRAC to mESCs. We identified 1,165 significant RNA-associated proteins in mESCs (Figure 6A; Table S1), including 466 previously uncharacterized, representing a 35% increase in the number of annotated mouse RNA-associated proteins (Figure 6B). This mESC dataset provides three advances.

First, the data can provide additional evidence to support assignment of novel RNPs. For example, many of the RNA-associated proteins identified in mESCs reflected equivalent RNA-associated proteins in human cells (Figure 6C), including the RFC complex, which specifically behaves as an RNP complex in both species (Figure 5B).

Second, this approach allowed the identification of cell-type-specific RNA-associated proteins. Indeed, we identified several mESC-specific RNA-associated proteins and these included several that have been previously implicated in stem cell function. For example, we identified the known pluripotency factor Sox2 (Figure 6D) and the polycomb repressor complex 2 subunit Jarid2 (Figure 6E), as RNA-associated proteins, consistent with previous reports (Cifuentes-Rojas et al., 2014; Fang et al., 2011; Kaneko et al., 2014).

Finally, the additional dataset allows us to cast a wider net in our search for novel RNPs. For example, among the RNA-associated proteins identified in mESCs were members of the centralspindlin complex, a heterotetramer consisting of Racgap1 and Kif23 and involved in cytokinesis (White and Glotzer, 2012; Yuce et al., 2005). Previously unknown to contain an RNA component, we identify Racgap1, Kif23 and the centralspindlin interaction partner Ect2 as significantly sensitive to RNase A treatment in mESCs (Figure 6F). In agreement with this mESC result, we observed a similar trend for this complex in human cells, showing conservation across species (Figure 6G). Our results suggest a physical interaction between the centralspindlin complex and RNA, thus informing a previous study that report Kif23 (ZEN-4 in *C. elegans*) as a positive regulator of RNP granule formation (Wood et al., 2016), as well as the localization of several RNA species to the midbody during cytokinesis (Clemson et al., 1996; Lécuyer et al., 2007; Zheng et al., 2010).

Together, these data demonstrate that the adaptability of DIF-FRAC to diverse systems will allow identification of conserved RNA-associated proteins and RNP complexes in diverse tissues and disease states across all domains of life.

### Advantages and Limitations of the DIF-FRAC Method

The field of protein RNA interactions has largely focused to date on identifying proteins that directly bind RNA. Our method is unique in its ability to identify larger modules of protein complexes that associate with RNA. It should be noted that our method is indirect in its ability to identify RNA binding. This indirect approach, however, not only allows us to identify RNP complexes but also allows us to identify them in a proteome-wide fashion using a very limited number of mass spectrometry experiments (~100 individual standard MS injections per DIF-FRAC experiment), 10–100 times less than required using an affinity purification strategy. Regardless of the nature of binding (direct versus indirect), we show the DIF-FRAC method strongly recapitulates previously identified RNA-binding proteins (Figures 1J and S2B–S2F). For example, DIF-FRAC identified 24 of the 25 RNA-binding proteins that have been identified by all 11 high-throughput studies (Table S3).

A further advantage of our method is the ability to discriminate between protein interactions that are and are not mediated by RNA. As mentioned above, most protein complex maps available do not consider protein RNA interactions, and moreover most experiments used to identify protein-protein interactions are done without an explicit step of removing nucleic acid. We have highlighted previously important protein interactions that are mediated by RNA (Drew et al., 2017b) and the need to identify interactions mediated by RNA. Here, we have developed a method with the ability to identify protein interactions that are mediated by RNA. Toward this, our method allows the categorization of complexes into classes including apo-stable and structural that define interactions as mediated solely by protein-protein interface or protein-RNA interfaces, respectively.

There are several potential limitations of our method that arise from the lysis requirement in our experimental procedure. First, during lysis there is a potential for gain of interactions of molecules that do not encounter each other in normal physiological settings (e.g., proteins from different organelles). Our experimental procedure does, however, mitigate this possibility by greatly decreasing the concentration of protein during lysis as well as during chromatography (~100-fold dilution). Second, our lysis conditions provide an environment that allows only stable interactions to be observed making transient interactions increasingly unlikely to be observed. Finally, our lysis conditions are optimized for soluble proteins and do not specifically enrich for membrane-bound proteins. Because of this bias, we likely miss potential RNA-associated membrane-bound proteins.

One final limitation of our approach involves the variation of protein abundance observed between the control sample and experiment sample. Other groups have cleverly approached this problem using a SILAC (stable isotope labeling by amino acids in cell culture) strategy (Kristensen et al., 2012). Unfortunately, a barrier to the SILAC approach occurs when adding an exogenous enzyme such as RNase A, because of the mixing step that will contaminate the control sample with the RNase A enzyme from the treated sample. In this work, we mitigate this variation in protein abundance through the use of statistics by calculating a rank ordered list that controls for the abundance of each protein. We therefore believe that our computational framework will be a powerful resource to be used for additional experiments in this regime.

### Conclusion

Here, we report the design, development, and application of a robust fractionation-based strategy to determine RNP complexes on a proteome-wide scale. We successfully used DIF-FRAC to identify 115 stable RNP complexes throughout the human interactome and applied DIF-FRAC to multiple cell types and species. Combining this with previous data, we generate a resource of the RNA-bound human proteome and demonstrate that upward of 20% of protein complexes contain an RNA component, highlighting the prevalence of RNP complexes in the cellular milieu. Together our results provide a valuable tool for researchers to investigate the role of RNPs in protein function and disease.

The DIF-FRAC methodology offers important advances over previous techniques to examine RNA-protein interactions. Specifically, interactions are probed proteome-wide in a native, whole-lysate sample using a strategy that is not reliant on labeling or cross-linking efficiency. We show that DIF-FRAC can be applied effectively to multiple cell types and organisms and has the potential to provide information on protein-RNA interactions in disease states. Furthermore, DIF-FRAC is a broadly applicable framework that can be extended to examine other large-scale proteomic changes in a system of interest.

We also introduce three classifications of RNP complexes (apo-stable, structural, and compositional) that provide a useful framework to organize the roles of RNAs in macromolecular complexes. Additionally, DIF-FRAC provides information on the biochemical characteristics (i.e., molecular weight, solubility) of RNP complexes in the presence and absence of RNA that offer clues to disease pathophysiology. We anticipate this technique to be a powerful tool to uncover the molecular mechanisms of RNA-related diseases. Overall, the DIF-FRAC method described and demonstrated here charts new territories in the cellular landscape of RNA-protein interactions. We have used DIF-FRAC to provide the first system-wide resource of human RNPs, providing a broadly applicable tool for studying cellular interactions and responses in multiple cell types and states.

## STAR⋆METHODS

### LEAD CONTACT AND MATERIALS AVAILABILITY

Further information and requests for resources and reagents should be directed to and will be fulfilled by the Lead Contact, Kevin Drew (kdrew@utexas.edu). This study did not generate new reagents.

### EXPERIMENTAL MODEL AND SUBJECT DETAILS

#### Human Cell Culture and Extract Preparation

HEK293T cells (ATCC CRL3216, sex: female) cultured in DMEM (GIBCO) supplemented with 10% (v/v) FBS (Life Technologies) were continually split over 7 days to give four 10-cm dishes of adherent cells. For the control fractionation sample, two 10-cm dishes of cells were harvested at 80%–100% confluence without trypsin by washing in ice cold phosphate buffered saline (PBS) pH 7.2 (0.75 mL; GIBCO) and placed on ice. Cells (approximately 0.1 g wet weight) were lysed on ice (5 min) by resuspension in Pierce IP Lysis Buffer (0.8 mL; 25 mM Tris-HCl pH 7.4, 150 mM NaCl, 1 mM EDTA, 1% NP-40 and 5% glycerol; Thermo Fisher) containing 1x protease inhibitor cocktail III (Calbiochem). The lysis step results in approximately a 10-fold dilution. The resulting lysate was clarified (17,000 g, 10 min, 4°C) and left at room temperature (30 min). The sample was filtered (Ultrafree-MC filter unit (Millipore); 12,000 g, 2 min, 4°C) to remove insoluble aggregates. RNase A treated samples were prepared on the same day in an identical manner, except RNase A (8 μL, 80 μg, Thermo Fisher, catalog #EN0531) was added after lysate clarification and the sample left at room temperature (30 min) before filtration.

#### Mouse Embryonic Stem Cell Culture

Gelatin adapted mouse J1 ES cells (ATCC® SCRC-1010, sex: male) were cultured in Dulbecco’s Modified Eagle’s Medium (DMEM, Life Technologies) containing 18% fetal bovine serum (FBS, Gemini), 50 U/mL of penicillin/streptomycin with 2 mM L-glutamine (Life Technologies), 0.1 mM non-essential amino acid (Life Technologies), 1% nucleosides (Sigma-Aldrich), 0.1 mM β-mercaptoethanol (Sigma-Aldrich), and 1,000 U/mL recombinant leukemia inhibitory factor (LIF, Chemicon). ES cells were plated on 15-cm dishes coated with 0.1% gelatin and incubated at 37°C and 5% CO_2_. Cells were passaged every 2 days. Lysis and RNase A treatment were done as described in the HEK293T protocol.

#### Erythrocyte Cell Preparation

Leukocyte-reduced red blood cells (RBCs) were obtained from an anonymous female donor and purchased from Gulf Coast Regional Blood Center (Houston, Texas). The RBCs used were kept at 4°C for either 7 days or 54 days depending on sample before lysis to ensure reticulocytes mature into RBCs. Prior to cell lysis, RBCs were washed with ice cold PBS (pH 7.4, GIBCO) for 3 times at 600 g for 15 min at 4°C. RBCs were then lysed in hypotonic solution (5 mM Tris-HCl, pH 7.4) containing protease and phosphatase inhibitors (complete, EDTA-free Protease Inhibitor Cocktail, Roche and PhosSTOP, Roche) with a ratio of 1 volume packed RBC: 5 volumes hypotonic solution. Hemolysate (soluble fraction of RBC lysate) was collected by centrifuging white ghosts (membrane fraction of RBC lysate) at 21,000 g for 40 mins at 4°C. Hemolysate was collected and stored at −80°C until further use. On the day of experiment, hemolysate was thawed and treated with Hemoglobind (Biotech Support Group) in order to remove hemoglobin from hemolysate. A total of 4–5 mg of total proteins were split into control and RNase A treated samples. The RNase sample was treated with RNase A as described in the protocol of RNase A treatment of lysate from HEK293T cells. Both samples were filtered (Ultrafree-MC filter unit (Millipore); 12,000 g, 2 min, 4°C) to remove insoluble aggregates prior to fractionation.

### METHOD DETAILS

#### Biochemical Fractionation Using Native Size-Exclusion Chromatography

All lysates were subject to size exclusion chromatography (SEC) using an Agilent 1100 HPLC system (Agilent Technologies, ON, Canada) with a multi-phase chromatography protocol as previously described (Havugimana et al., 2012). Soluble protein (1.25 mg, 250 μL) was applied to a BioSep-SEC-s4000 gel filtration column (Phenomenex) equilibrated in PBS, pH 7.2 (HEK293T and mESC lysate) or pH 7.4 (erythrocytes) at a flow rate of 0.5 mL min^−1^. Fractions were collected every 0.375 mL. The resulting dilution from input lysate is approximately 100-fold. The elution volume of molecular weight standards (thyroglobulin (M_r_ = 669 kDa); apoferritin (M_r_ = 443 kDa); albumin (M_r_ = 66 kDa); and carbonic anhydrase (M_r_ = 29 kDa); Sigma) was additionally measured to calibrate the column (Figure 1B).

#### Mass Spectrometry

Fractions were filter concentrated to 50 μL, denatured and reduced in 50% 2,2,2-trifluoroethanol (TFE) and 5 mM tris(2-carboxyethyl) phosphine (TCEP) at 55°Cfor45 minutes, and alkylated in the dark with iodoacetamide (55 mM, 30 min, RT). Samples were diluted to 5% TFE in 50 mM Tris-HCl, pH 8.0, 2 mM CaCl_2_, and digested with trypsin (1:50; proteomics grade; 5 h; 37°C). Digestion was quenched (1% formic acid), and the sample volume reduced to ~100 μL by speed vacuum centrifugation. The sample was washed on a HyperSep C18 SpinTip (Thermo Fisher), eluted, reduced to near dryness by speed vacuum centrifugation, and resuspended in 5% acetonitrile/ 0.1% formic acid for analysis by LC-MS/MS.

Peptides were separated on a 75 μM × 25 cm Acclaim PepMap100 C-18 column (Thermo) using a 3%–45% acetonitrile gradient over 60 min and analyzed online by nanoelectrospray-ionization tandem mass spectrometry on an Orbitrap Fusion or Orbitrap Fusion Lumos Tribrid (Thermo Scientific). Data-dependent acquisition was activated, with parent ion (MS1) scans collected at high resolution (120,000). Ions with charge 1 were selected for collision-induced dissociation fragmentation spectrum acquisition (MS2) in the ion trap, using a Top Speed acquisition time of 3 s. Dynamic exclusion was activated, with a 60 s exclusion time for ions selected more than once. MS from HEK293T cells was acquired in the UT Austin Proteomics Facility.

#### Construction and Sequencing of RNA-Seq Libraries of DIF-FRAC Samples

Fractions from a biological replicate SEC separation corresponding to higher molecular weight species (approximately >1.5 MDa; fractions 16–23 in Figure 1B) were analyzed by total RNA sequencing. Total RNA was isolated from each fraction (0.375 mL) by addition of Trizol (1.125 mL; Thermo Fisher) and the sample (1.4 mL) was transferred to a Phasemaker tube (Thermo Fisher). Total RNA was extracted following the protocol supplied by the manufacturer and further cleaned up using a RNeasy MinElute Cleanup Kit (QIAGEN). RNA integrity number (RIN) was measured using an Agilent Bioanalyzer and samples were ribo-depleted using a using a RiboZero Gold (Human/Mouse/Rat) kit (Illumina) to remove rRNAs. RNA libraries were prepared for sequencing according to vendor protocols using NEBNext R Small RNA Library Prep Set for Illumina R (Multiplex Compatible), Cat #E7330L, according to the protocol described by Podnar et al. (2014). RNA was fragmented using elevated temperature in carefully controlled buffer conditions to yield average fragment sizes of 200 nucleotides. These fragments were directionally ligated to 5′ and 3′ adaptors so that sequence orientation is preserved throughout sequencing. Reverse transcription and PCR were performed to complete the DNA sequencing libraries, which were sequenced using an Illumina NextSeq 500 instrument (75-nt single reads) at the Genomic Sequencing and Analysis Facility at the University of Texas at Austin.

#### *S. cerevisiae* RFC Purification

RFC was purified as previously described (Finkelstein et al., 2003; Kim et al., 2017). Briefly, full-length *S. cerevisiae* RFC was expressed in BL21(DE3) ArcticExpress (Agilent) *E. coli* co-transformed with pLant2b-RFC-AE (pIF117) and pET11-RFC-BCD (pIF116). RFC was subsequently purified by SP and Q (GE Healthcare) ion exchange chromatography. Protein concentration was determined by comparison to a BSA titration curve using Coomassie-stained SDS-PAGE.

#### Electrophoretic Mobility Shift Assay (EMSA)

Oligonucleotide constructs were based on an earlier description (Kobayashi et al., 2006). Each of the four-nucleic acid substrates were radiolabeled with [γ−^32^P]-ATP using T4 Polynucleotide Kinase (NEB). Free nucleotide was removed using G-25 MicroSpin columns (GE Healthcare). Oligonucleotides were subsequently heated to 75°C and slowly cooled to room temperature to allow proper annealing. 1 nM oligonucleotide and various concentrations of RFC (0 to 256 nM) were incubated for 15 minutes at room temperature in a buffer containing 25 mM Tris-HCl [pH 7.5], 50 mM NaCl, 2 mM MgCl_2_,2 mM DTT, and 0.1 mg/mL BSA. Reactions were quenched with 6x loading dye (10 mM Tris-HCl [pH 7.6], 60% glycerol, 60 mM EDTA, 0.15% [w/v] Orange G) and subsequently separated by native acrylamide gel electrophoresis. Gels were dried on Zeta-Probe Membrane (Bio-Rad) at 80°C for two hours. Bands were visualized by a Typhoon FLA 7000 phosphorimager (GE Healthcare). Binding was quantified using FIJI (Schindelin et al., 2012). Subsequent data were fit to a hyperbolic equation to determine the k_D_ for oligonucleotide binding.

#### Oligonucleotides used

**Table:** 

Name	Sequence
dsDNA	5′ - CTC GAG GTC GTC ATC GAC CTC GAG ATC A – 3′
dsRNA	5′ - rCrUrC rGrArG rGrUrC rGrUrC rArUrC rGrArC rCrUrC rGrArG rArUrC rA – 3′

Calculated k_D_ from fitting to hyperbolic equation (Bound = (v*[E])/(k_D_+[E])), where “[E]” is the concentration of the enzyme, and “v” and “k_D_” are solved by linear regression.

### QUANTIFICATION AND STATISTICAL ANALYSIS

#### Protein Identification

Prior to protein identification, human and mouse proteomes were downloaded from UniProt website (Apweiler et al., 2004). Raw formatted mass spectrometry files were first converted to mzXML file format using MSConvert (http://proteowizard.sourceforge.net/tools.shtml) and then processed using MSGF+ (Kim et al., 2017), X! TANDEM (Craig and Beavis, 2004) and Comet (Lingner et al., 2011) peptide search engines with default settings. MSBlender (Kwon et al., 2011) was used for integration of peptide identifications and subsequent mapping to protein identifications. A false discovery rate of 1% was used for peptide identification. Protein elution profiles were assembled using unique peptide spectral matches for each protein across all fractions collected.

#### DIF-FRAC Score and P Value Significance Calculation

In order to determine the significance of a protein’s sensitivity to RNase A treatment, we compare the protein’s control elution profile to its RNase A treated elution profile as schematized in S2A. Specifically, we first calculate the L1-norm of the two elution profiles (Equation 1).

(1) $$D_{p}=\sum_{i=1}^{N}\left|X_{p,i}-Y_{p,i}\right|$$

Where *N* represents the total number of fractions collected and *p* represents an individual protein. *X* and *Y* represent abundance matrices of control and experiment (RNase A treated) respectively. We next normalize *D*_*p*_ by the total abundance seen for protein *p* in both the control and experiment (Equation 2).

(2) $$D_{p}^{norm}=\frac{D_{p}^{2}}{\sum_{i=1}^{N}X_{p,i}+\sum_{i=1}^{N}Y_{p,i}}$$

We observed *D*^*norm*^ is biased by high abundance proteins and we therefore evaluate significance of a protein’s sensitivity to RNase A treatment by comparing to a background of proteins with similar abundance. Specifically, we create a distribution of *D*^*norm*^ from proteins in a window surrounding protein *p* and have not been annotated as RNA-associated proteins in the literature (Equation 3). See Figure S2A for schematic.

(3) $$W_{p}=D_{p+s}^{norm},\ldots ,D_{p}^{norm},\ldots ,D_{p-s}^{norm}$$

where *s* is a window size of 100 and unannotated RNA-associated proteins are in order of abundance.

We posit that the proteins in distribution, *W*_*p*_, is a mixture of unannotated RNA-associated proteins as well as non-RNA-associated proteins. In order to evaluate significance of a protein’s *D*^*norm*^ being greater than what is expected by non-RNA binders, we model the distribution *W*_*p*_ using a two component Gaussian mixture model (GMM). To ensure an accurate model fit we evaluate our GMM fit using three criteria (Equation 4). First, we calculate the Bayesian Information Criterion (BIC) for both the two component GMM and a one component GMM and ensure the two component GMM has a lower BIC (Equation 4a). Second, we ensure the component with the lowest mean μ (i.e., non-RNA-associated component) has the largest weight (Equation 4b). Finally, we ensure the largest component weight is greater than a given weight threshold *t*_*weight*_ (Equation 4c). *t*_*weight*_ can be estimated by the expected fraction of non-RNA binders in the proteome. In practice we set *t*_*weight*_ to be between 0.6 and 0.75.

(4a) $$BIC_{2-component}\leq BIC_{1-component}$$

(4b) argmin=argmaxμweight

(4c) $$\text{max}\geq t_{weight}\;weight$$

If all three criteria are passed the lowest mean component of the two component GMM is used, otherwise the one component is used (Equation 5).

(5) $$GMM_{p}=\left\{ \begin{array}{l} GMM_{2-component,}\;\text{if}\;\text{criteria}\;\text{are}\;\text{met}\;\left(\text{equation 4}\right)\\ GMM_{1-component,}\;\text{otherwise} \end{array}\right.$$

We next calculate the Z-score of protein *p*’s *D*^*norm*^ score relative to the non-RNA-associated component of GMM_p_ (Equation 6).

(6) $$Z_{p}=\frac{D_{p}^{norm}-\mu_{GMM_{p}}}{\sigma_{GMM_{p}}}$$

where *μ*_*GMMp*_ and *σ*_*GMMp*_ are the mean and standard deviation of the first component of GMM_p_ respectively.

Finally, we calculate a p value of *Z*_*p*_ using the normal distribution survival function and then false discovery correct p values across all proteins using the Benjamini/Hochberg correction (Benjamini and Hochberg, 1995). RNA-associated proteins were considered significant at a 0.05 FDR corrected p value.

It is also worth mentioning our method is robust to poorly supported protein identifications. Specifically, the p value calculation is based on a sliding abundance window which in effect penalizes poorly observed proteins. In practice, RNA-associated proteins identified by our approach have a minimum mean abundance of 12 peptide spectral matches across control and RNase A samples.

#### RNA-Binding Annotations, Overlap Comparisons, and Score Performance Analysis

Low throughput RNA binding annotations were defined as proteins with Gene Ontology (Ashburner et al., 2000) “RNA binding” annotations limited to those with evidence codes: EXP, IDA, IPI, IMP, IGI, IEP, TAS, NAS, or IC. In addition, proteins with “ribonucleo-protein” in their UniProt keywords were also included. Direct high throughput RNA binding annotations were primarily collected from Table S2 in Hentze et al. (2018). In addition, we gathered more recent direct high throughput datasets from Bao et al., (2018), Huang et al. (2018), Queiroz et al. (2019), and Trendel et al. (2019) and indirect methods from Brannan et al. (2016) and Caudron-Herger et al. (2019).

To estimate the coverage of identified RNA-associated proteins by the DIF-FRAC method independent of cell type and machine setup, the Venn diagrams in Figures 6B and S3N report only proteins with mean abundance > = 10, where mean abundance is the average peptide spectral matches identified in the control and RNase A treated HEK293T cells. To compare directly the RNA-associated proteins identified in the high throughput sets to the DIF-FRAC method, Venn diagrams in Figures S3A–S3M report all proteins.

To calculate Precision versus Neg Ln p value plots (Figure 1I; Figure S2C), we first added a pseudocount (+1e-308) to DIF-FRAC p values and then applied −1*ln(p value) where ln is the natural log. Precision is defined as TP/AP, where TP (true positives) is defined as proteins annotated as either high throughput or low throughput RNA binding (see above) and a Neg Ln p value greater than a given value. AP (all predictions) is defined as any protein with a Neg Ln p value greater than a given value. To calculate Precision versus Recall plots (Figures S2B and S2D), precision is defined above and recall is defined as TP/AKP where TP is true positives and AKP (all known positives) is defined as proteins annotated as either high throughput or low throughput RNA binding.

#### Classification of DIF-FRAC Elution Profiles

To calculate the amount a protein shifts upon RNase A treatment, we calculate the average fraction a protein is observed weighted by the PSMs observed in each fraction. The difference between the weighted average of the treated and untreated elution profiles provides the total shift amount. A protein’s shift in elution from a high molecular weight to a low molecular weight results in a negative shift value whereas a shift from low molecular weight to high molecular weight corresponds to a positive value.

To calculate the amount a protein’s abundance changes upon RNase A treatment, we calculate the difference of a protein’s total PSMs observed in the untreated and treated samples. We further normalize this value by dividing by the sum of the total PSMs from both samples. This results in a value between 1.0 and −1.0 where a positive value corresponds to an increase in abundance upon RNase A treatment and a negative value corresponds to a decrease in abundance upon RNase A treatment.

#### Assembly of RNP Complexes

We define the global set of RNP complexes by first creating a combined non-redundant set of CORUM (Ruepp et al., 2010) and hu.MAP (Drew et al., 2017a) complexes (Jaccard coefficient < 1.0). For every complex in this global set we tested if > 50% of the protein subunits were 1) identified as an RNA-associated protein by DIF-FRAC (p value > 0.05), 2) annotated by high throughput methods or 3) annotated by low throughput methods (see above for description of annotations). RNP Select complexes are defined as complexes whose protein subunits co-elute in the DIF-FRAC control sample (> 0.75 average Pearson correlation coefficient among subunits) and > 50% of subunits have a DIF-FRAC p value > 0.5. Here we relax the p value threshold with the rationale that multiple co-complex subunits passing this threshold provides additional support for RNA association.

#### RNA Hairpin Pull-down Reanalysis

Raw files from Treiber et al. were downloaded from PRIDE (PXD004193) and processed as described above using MSBlender. Distributions in Figures S4C–S4D were calculated based on the mean peptide spectral matches (PSM) of each protein in a given complex. The RNP and RNP Select complexes are as defined above. The RNP_Novel complexes are RNP complexes which had previously had < 50% subunits annotated as RNA associated by high throughput or low throughput methods. NonRNP complexes are complexes with < 50% subunits annotated as RNA associated by high throughput, low throughput or DIF-FRAC methods. To determine statistical significance in terms of difference among distributions, we used the scipy (Jones et al., 2015) stats library Mann-Whitney U test.

#### RNA-Seq Analysis

After performing quality control on the sequencing fastq files using FastQC (www.bioinformatics.babraham.ac.uk/projects/fastqc/), 3′ adaptor contamination was removed using Cutadapt (v1.10) (Martin, 2011). Alignment of the 8 RNA fraction datasets was then performed with the Hisat2 transcriptome-aware aligner (v2.1.0) (Kim et al., 2015), against a Hisat2 reference index built using GRCh38/hg38 primary assembly genome fasta from Gencode (v27, Ensembl release 90) (Harrow et al., 2012) annotated with the corresponding v27 GTF (General Transfer Format) annotations. The Hisat suite Stringtie program (v1.3.3b) (Pertea et al., 2016) was used to quantify gene-level expression from the alignment files. TPM (Transcripts Per Million), a sequencing-depth-normalized estimate of reads mapping to the gene, was used for further analysis.

### DATA AND CODE AVAILABILITY

#### Data Deposition

Proteomics data are deposited in Pride with accessions PRIDE: PXD015406, PRIDE: PXD014820, and PRIDE: PXD014607. RNA-seq data are deposited in Gene Expression Omnibus (GEO: GSE137651).

#### Code Repository

Source code is freely available on GitHub: https://github.com/marcottelab/diffrac

### ADDITIONAL RESOURCES

Website providing online access of RNP complexes can be found here: http://rna.proteincomplexes.org/

## Supplementary Material

1

2

3

4

## Figures and Tables

**Table 1.** Stable RNPs Identified by DIF-FRAC

Gene Names	Complex Name	Function	Soluble without RNA?^a^	Disease Links	CORUM/hu.MAP^b^	rna.MAP ID	DIF-FRAC Plot	RNP Class^c^	References
CLASP1	N/A	microtubule binding	yes	N/A	no/yes	3807		apo-stable	Efimov et al., 2007
CLASP2		microtubule dynamics							
DAXX	DAXX-TP53 complex	transcription repression	yes	pancreatic neuroendocrine tumors	yes/no	4518		apo-stable	Zhao et al., 2004
TP53				glioblastoma multiforme					Dyer et al., 2017
				adrenocortical tumors					
DRG1	Drg1/Dfrp1 complex	microtubule binding	yes	lung adenocarcinoma	no/no	4096		apo-stable	Lu et al., 2016
ZC3H15		microtubule polymerase							Schellhaus et al., 2017
		GTPase							Ishikawa et al., 2009
BOD1L1	SET1A/SET1B complexes	histone methyltransferase	yes	Fanconi anemia	yes/yes	2005, 3307		apo-stable	Vedadi et al., 2017
SETD1A		transcription regulation		mixed-lineage leukemia					Higgs et al., 2015
CXXC1*^,d^									Brown et al., 2017
ASH2L									
RBBP5									
WDR5									
NIPSNAP1*	N/A	vesicular transport	no	inflammatory pain	no/yes	5822		apo-stable	Okuda-Ashitaka et al., 2012
NIPSNAP2*									Yamamoto et al., 2017
RPA1	replication protein A complex	single-stranded DNA binding	yes	Werner syndrome	yes/yes	3204		apo-stable	Machwe et al., 2011
RPA2*		DNA metabolism							Fan and Pavletich, 2012
RPA3*^,d^									
FLII	FLII-LRRFIP1 complex	transcriptional activation	yes	prostate cancer	no/yes	3626		apo-stable	Wilson et al., 1998
LRRFIP1		actin binding							Wang et al., 2016
BAZ1A*	WCRF complex	chromatin remodeling	No	intellectual disability	yes/no	2105		compositional	Bochar et al., 2000
SMARCA5									Zaghlool et al., 2016
MICU1*^,d^	MICU1-MICU2 heterodimer	calcium ion transport	yes	myopathy with extrapyramidal signs	yes/yes	4318		structural	Logan et al., 2014
MICU2*^,d^									
NOC4L	N/A	ribosome processing and biogenesis	no	recurrent pregnancy loss	no/yes	4220		structural	Suzuki et al., 2018
NOP14									
SFPQ	PSF-p54(nrb) complex	splicing factor	yes	intellectual disability	yes/yes	327		apo-stable	Bladen et al., 2005
NONO		DNA recombination							Mircsof et al., 2015
RRP12	N/A	rRNA processing	no	N/A	no/yes	5795		structural	Zemp et al., 2009
RIOK2									
H1FX									
SAP18	N/A	SAP18 is involved in RNA processing and splicing	no	N/A	no/yes	6027		structural	Davis et al., 2010
NKTR									
CCDC9		NKTR is involved in protein peptidyl-prolyl isomerization							
LARP4	N/A	translation regulation	yes	N/A	no/yes	3327		apo-stable	Yang et al., 2011
LARP4B									Schäffler et al., 2010
XRCC5	Ku antigen complex	DNA damage and repair	yes	systemic lupus erythematosus	yes/no	2930		apo-stable	Spagnolo et al., 2006
XRCC6									
SAMM50*^,d^	N/A	protein transport	yes	N/A	no/yes	1450		apo-stable	
MTX3*^,d^									
MTX2*									
SLC25A5	prohibitin	apoptosis	yes	N/A	yes/no	204		apo-stable	Kasashima et al., 2006
VDAC2									
PHB									
HAX1*									
PHB2*									
TPP2	tripeptidyl-peptidase II	serine protease	yes	muscle wasting	yes/no			apo-stable	Schönegge et al., 2012
				obesity					Rockel et al., 2012
				cancer					

* Previously unreported RNA-associated proteins identified by DIF-FRAC (see Table S1).

a Insolubility in the absence of RNA is inferred by an increase in apparent molecular weight of the complex upon RNA digestion or a complete disappearance of signal. This is consistent with the RNP’s being solubilized by RNA, as suggested by Maharana et al. (2018).

b Evidence for all or some protein complex subunits interacting in CORUM or hu.MAP.

c RNP classes apo-stable, structural, and compositional, as described in Figure 4.

d Previously unreported RNA-associated proteins that are above the 5% FDR cutoff.

**KEY RESOURCES TABLE:** 

REAGENT or RESOURCE	SOURCE	IDENTIFIER
Biological Samples		
Red Blood Cells	Gulf Coast Regional Blood Center	E6401
Chemicals, Peptides, and Recombinant Proteins		
RFC	Kim et al. Sci Rep 2017 https://doi.org/10.1038/s41598-017-01984-x	N/A
Deposited Data		
RNA Hairpin Pulldowns (Treiber et al., 2017)	PRIDE Archive	PRIDE: PXD004193
HEK293 DIFFRAC data	PRIDE Archive (this paper)	PRIDE: PXD015406
HEK293 DIFFRAC replicate data	PRIDE Archive (this paper)	PRIDE: PXD014820
Mouse Embryonic Stem Cell DIFFRAC data	PRIDE Archive (this paper)	PRIDE: PXD014607
RNA-seq data	Gene Expression Omnibus (this paper)	GEO: GSE137651
Experimental Models: Cell Lines		
HEK293T	ATCC	CRL3216
Mouse J1 ES	ATCC	SCRC-1010
Oligonucleotides		
dsDNA	Kobayashi et al., 2006	N/A
dsRNA	Kobayashi et al., 2006	N/A
Software and Algorithms		
DIF-FRAC analysis software	https://github.com/marcottelab/diffrac	N/A
MSBlender	https://github.com/marcottelab/MSblender	N/A

## Highlights

- DIF-FRAC systematically discovers ribonucleoprotein complexes
- Identification of 1,428 RNP complexes (20% of known complexes)
- RNP complexes fall into three categories: Apo-stable, structural, and compositional
- Discovery of previously unknown roles for RNA in known protein complexes
