# Supplementary material for: Systematic Discovery of Endogenous Human Ribonucleoprotein Complexes
Source: Cell Rep. Author manuscript; Available in PMC 2019 Nov 22. (PMC6873818; doi:10.1016/j.celrep.2019.09.060)
Supplement: 1 [file NIHMS1542672-supplement-1.pdf]

Cell Reports, Volume *29*

Supplemental Information

Systematic Discovery of Endogenous Human Ribonucleoprotein Complexes

Anna L. Mallam, Wisath Sae-Lee, Jeffrey M. Schaub, Fan Tu, Anna Battenhouse, Yu Jin Jang, Jonghwan Kim, John B. Wallingford, Ilya J. Finkelstein, Edward M. Marcotte, and Kevin Drew

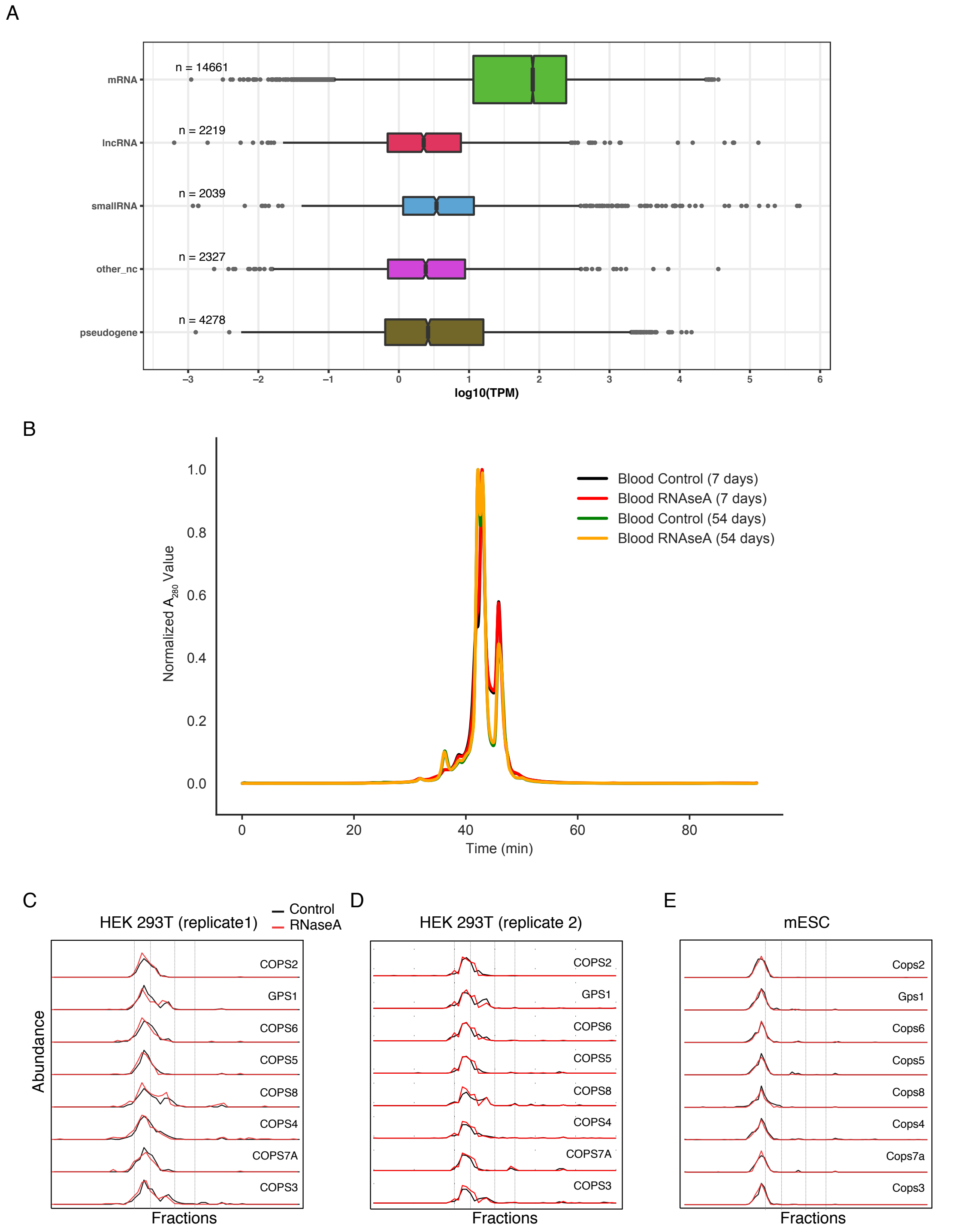

Figure S1: DIF-FRAC accesses a diverse RNA landscape and is specific to RNP complexes. Related to Figure 1. (A) Box plots show the RNA abundance of mRNA, lncRNA, small RNA, other ncRNA, and pseudogenes in control fractions 16-23 of HEK 293T cell lysate (TPM = Transcripts Per Million). Boxes indicate median (inner joint), first quartile (left) and third quartile (right). Lines indicate 1.5 interquartile range. Dots indicate outliers. (B) Comparison of negative control separations of 7-day matured erythrocyte lysate under control (black) and RNase A treated (red) conditions vs. 54-day matured erythrocyte lysate under control (green) and RNase A treated (orange) conditions as monitored by bulk SEC chromatography absorbance profiles at $A_{280.}$ No substantial change in absorbance signal is observed as a function of maturation time. (C-E) Non-RNA-associated complexes are insensitive to RNase A treatment. DIF-FRAC elution profiles show subunits of the negative control non-RNA-associated COP9 signalosome complex

(M_r ~500 kDa (Oron et al., 2002)) in control (black) and RNase A treated (red) for (C) HEK293T lysate, (D) HEK293T replicate and (E) mESC do not shift upon RNase A treatment. Abundance represents count of unique peptide spectral matches. Vertical dotted lines represent protein standards described in Figure 1.

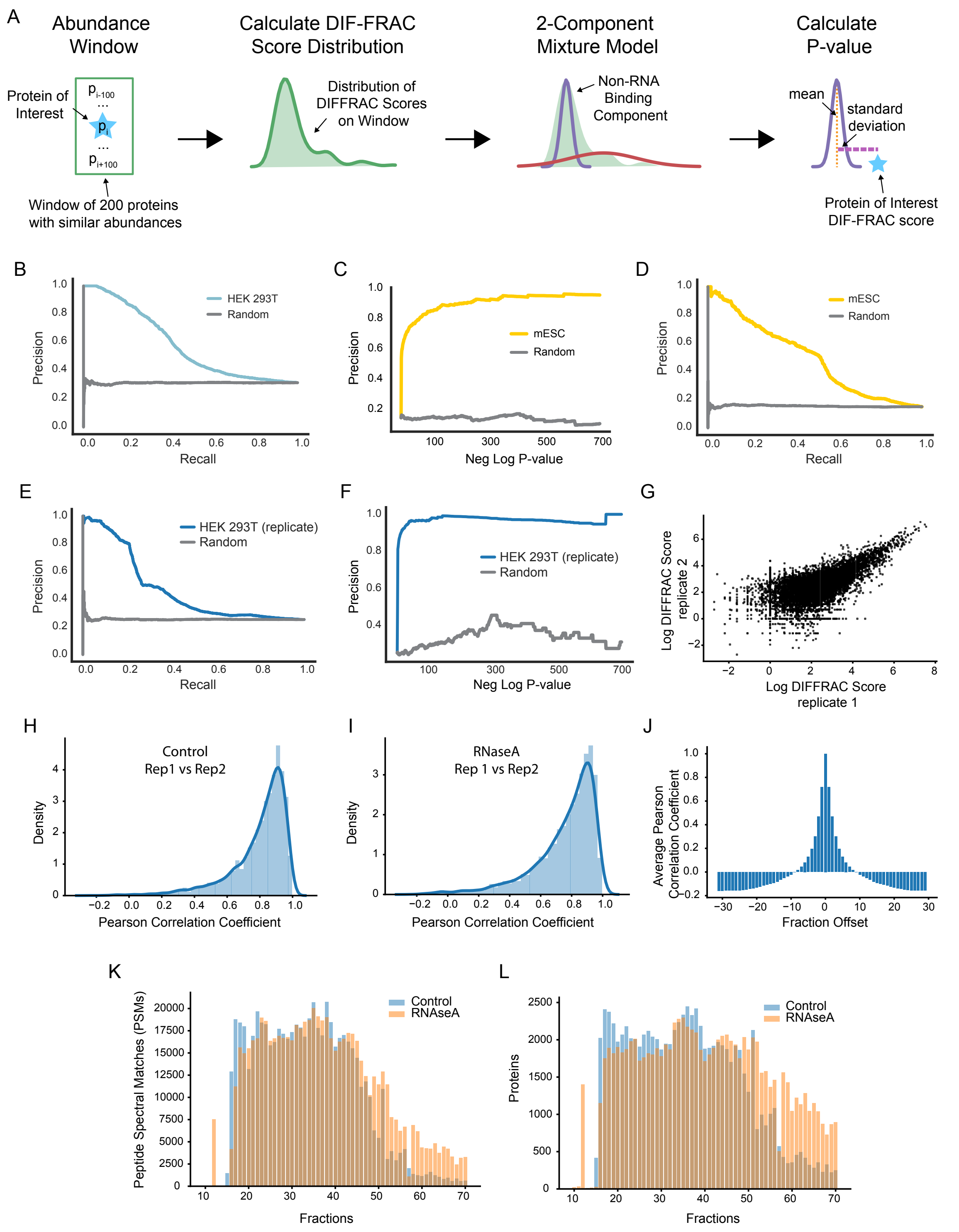

Figure S2: DIF-FRAC Score accurately discriminates between RNA-binding proteins and non-binders. Related to Figure 1. (A) Workflow to calculate abundance corrected P-values for each protein's DIF-FRAC score. Proteins are ranked according to abundance and a window of +/- 100 proteins is used to calculate a DIF-FRAC score distribution. A two-component Gaussian mixture model is then used to identify the non-RNA binding component in the distribution. Finally, the DIF-FRAC score of the protein of interest is compared to the non-RNA binding distribution component to test the null hypothesis and a P-value is calculated. (B) Precision recall analysis shows the DIF-FRAC Score recalls a substantial number of

known RNA-binding proteins in HEK 293T cells. (C) High DIF-FRAC P-values have high precision in recovering known RNA-binding proteins in mouse embryonic stem cells. (D) Precision recall analysis shows the DIF-FRAC Score recalls a substantial number of known RNA-binding proteins in mESC. (E) Precision recall analysis shows the DIF-FRAC Score is robust and reproducible on a replicate of HEK 293T cells. (F) High DIF-FRAC P-values have high precision in replicate of HEK 293T cells. (G) DIF-FRAC Score for each protein identified in both HEK 293T cell replicates shows a high degree of agreement. (H) Distribution of Pearson correlation coefficients of elution profiles across replicate control experiments (PSM >=10). (I) Distribution of Pearson correlation coefficients of elution profiles across replicate RNAse A treated experiments (PSM >=10). (J) Average Pearson correlation coefficient of fractional offsets shows nearby fractions are highly correlated. (K) Total PSMs per fraction shows a shift from high molecular weight fractions to lower molecular weight fractions upon RNAse A treatment. (L) Total proteins identified per fraction again shows a shift from high molecular weight fractions to lower molecular weight fractions upon RNAse A treatment.

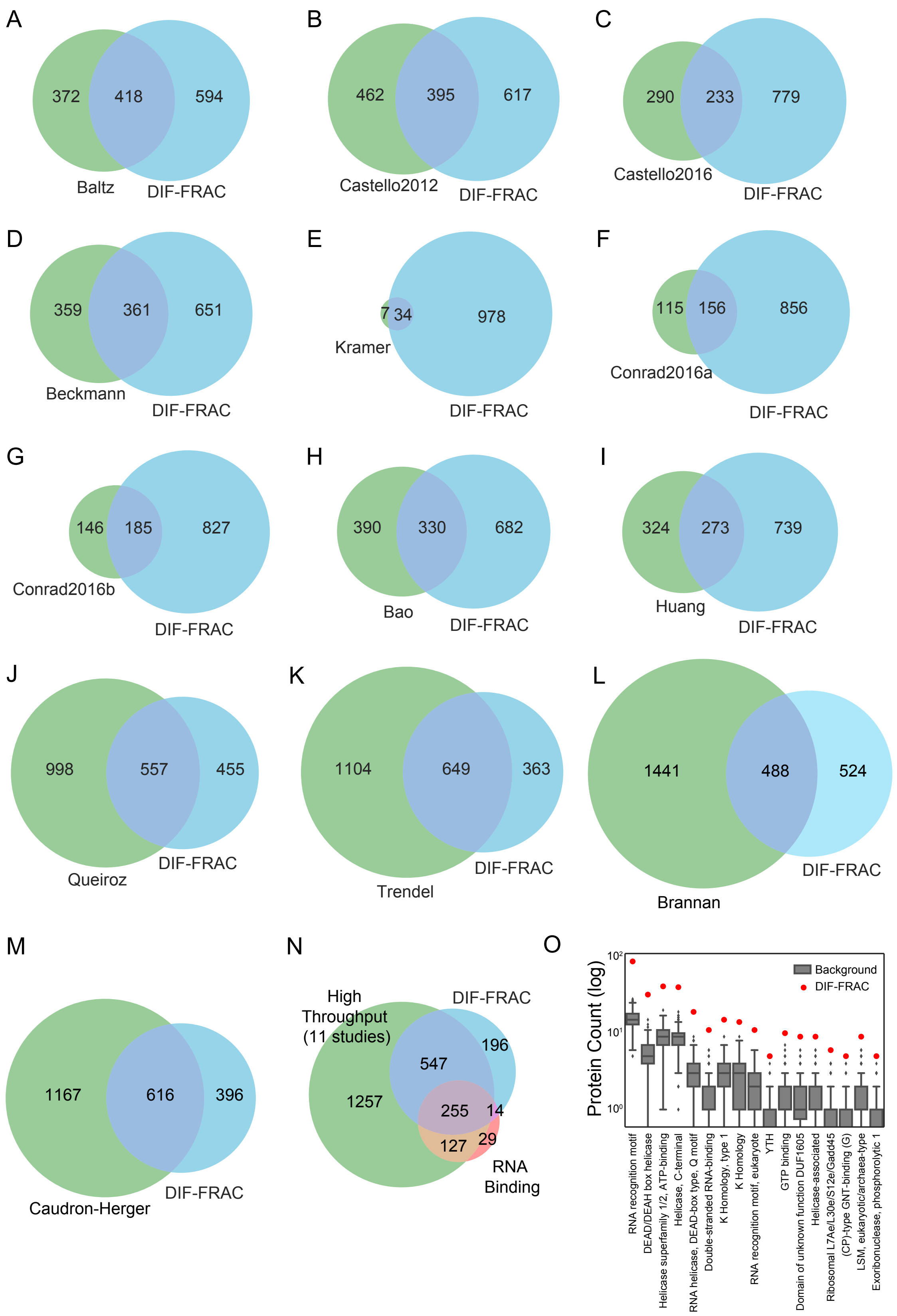

Figure S3: DIF-FRAC RNA-associated proteins show substantial overlap with other high-throughput studies. Related to Figure 1. (A-M) Venn diagrams show overlap of DIF-FRAC RNA-associated proteins from HEK 293T cells (blue) with 13 high-throughput RNA association studies (green) (Baltz et al., 2012; Bao et al., 2018; Beckmann et al., 2015; Brannan et al., 2016; Castello et al., 2012, 2016; Caudron-Herger et al., 2019; Conrad et al., 2016; Hentze et al., 2018; Huang et al., 2018; Kramer et

al., 2014; Queiroz et al., 2019; Trendel et al., 2019). (N) Venn diagram shows overlap of DIF-FRAC RNA-associated proteins from HEK 293T cells (blue), annotated RNA Binding proteins (red), and combined set of high throughput RNA association studies (green). (O) Enrichment of RNA binding structural motifs in DIF-FRAC-identified RNA-associated proteins from HEK 293T cells.

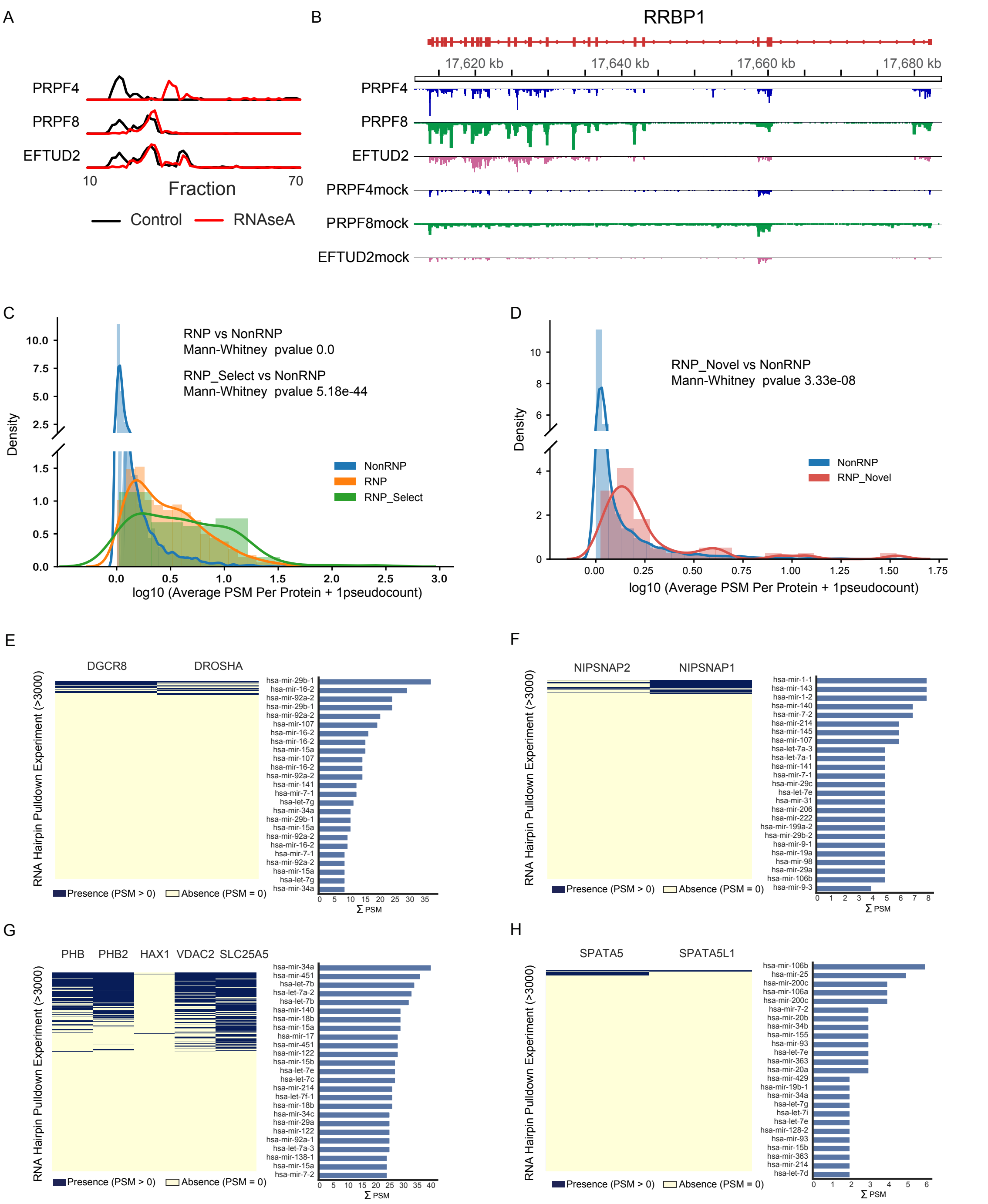

Figure S4: DIF-FRAC identified RNP complexes are supported by external datasets. Related to Figure 2. (A-B) DIF-FRAC identified RNP has consistent binding *in vivo*. (A) Elution profiles of U4/U6-U5 tri-snRNP complex subunits show sensitivity upon RNAse A treatment. (B) eCLIP data from ENCODE(Van Nostrand et al., 2016) shows subunits with coordinated binding of RRBP1 mRNA in HepG2 cells. All traces are scaled equivalently. ENCODE file accessions used: ENCFF224ZJZ, ENCFF095PCI, ENCFF206TXR, ENCFF698WXG, ENCFF974FWQ and ENCFF348XHW. (C-H) RNP Complexes are identified in RNA hairpin pulldown experiments. (C) Distribution of RNP and RNP Select complexes identified in RNA hairpin pulldown experiments. RNP and RNP Select distributions are observed more frequently (right shift) than NonRNP complexes as expected. (D) Novel RNP complexes are observed more frequently in pulldown experiments than NonRNP complexes suggesting some novel RNP

complexes interact with hairpins. (E-H) Left panel represents presence/absence plot of complex identified in RNA hairpin pulldown experiments as positive control. Right panel shows top 25 hairpin experiments by sum PSM. (E) Microprocessor complex (positive control). (F) Novel RNP NIPSNAP1/2 complex. (G) Novel RNP prohibitin-2 complex. (H) Novel RNP SPATA complex.

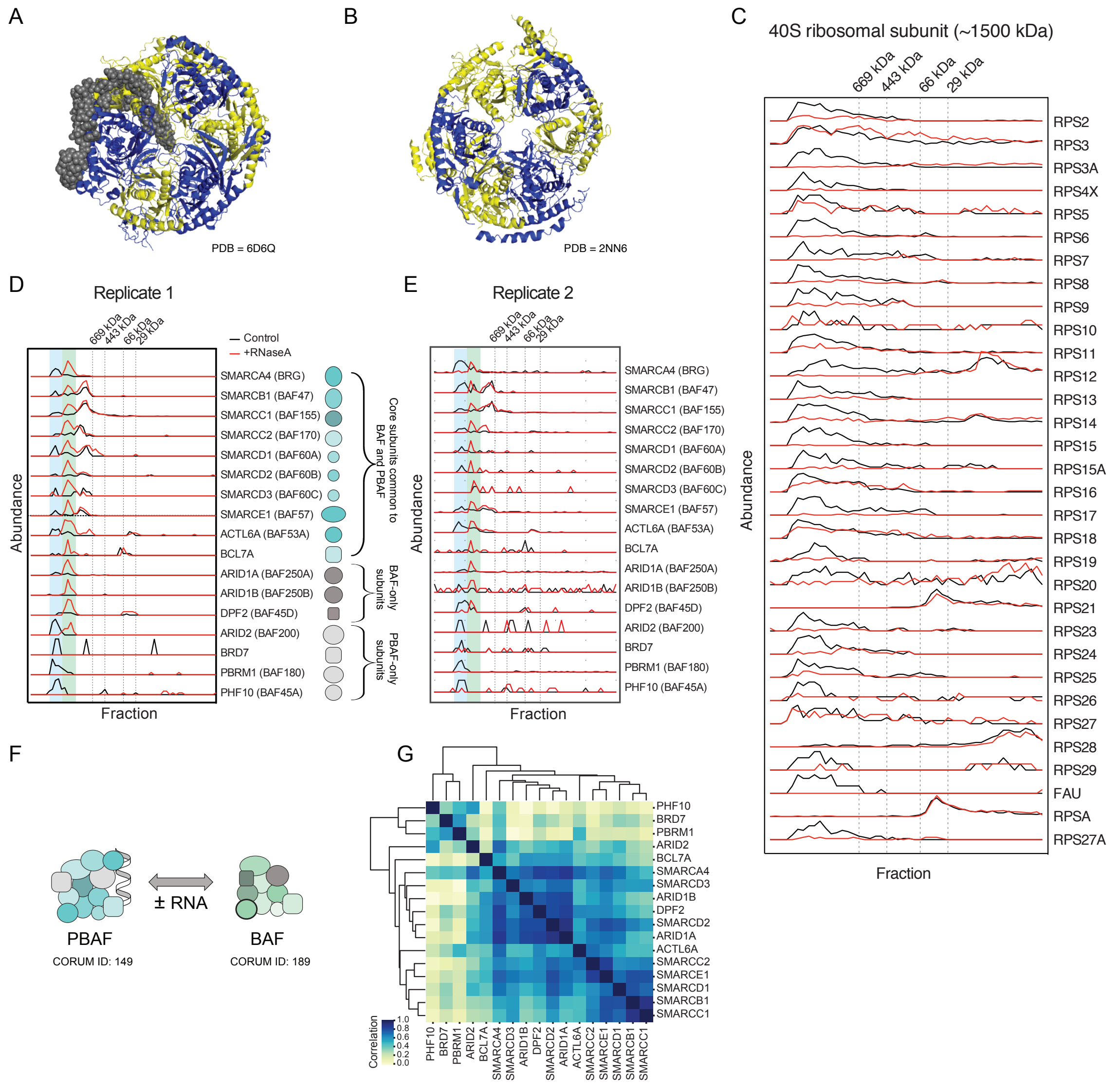

Figure S5: DIF-FRAC classifies RNP complexes into 3 classes. Related to Figure 3. (A-B) Atomic structure of apo-stable RNP complex demonstrate RNA is peripheral to the complex. (A) Structure of exosome complex protein subunits (blue/yellow) with bound RNA molecule (grey) (B) Structure of exosome complex protein subunits (blue/yellow) crystallized without RNA. This demonstrates protein interactions are responsible for the stability of the exosome complex. (C) Elution profiles of the 40S ribosomal subunits demonstrate it is destabilized upon RNA degradation ('structural' RNP complex). (D-G) DIF-FRAC classifies human BAF and PBAF complexes as compositional RNPs. (D) Elution profiles of annotated human PBAF (blue shading) and BAF (green shading) complexes demonstrate core subunits common to both complexes coelute in both control and RNase A treated samples, but at different molecular weights. Literature annotated PBAF-only subunits (light grey) coelute with core subunits only in the control sample, while literature annotated BAF-only subunits (dark grey) coelute with the core subunits as a lower molecular weight complex only when RNA is degraded. Together, these elution profiles suggest that PBAF is an RNP complex, but the BAF complex does not associate with RNA. (E) Elution profiles of PBAF and BAF complexes in replicate DIF-FRAC experiment. Colors the same as in A. (F) Cartoon of known PBAF and BAF complexes as defined by CORUM id 149 and 189 respectively with RNA molecule

associated with PBAF. (G) Clustergram of Pearson correlation coefficient from replicate 1 and 2 elution profiles shows clusters are consistent with known annotations with the exception of ARID2 which clusters between BAF-only and PBAF-only subunits.

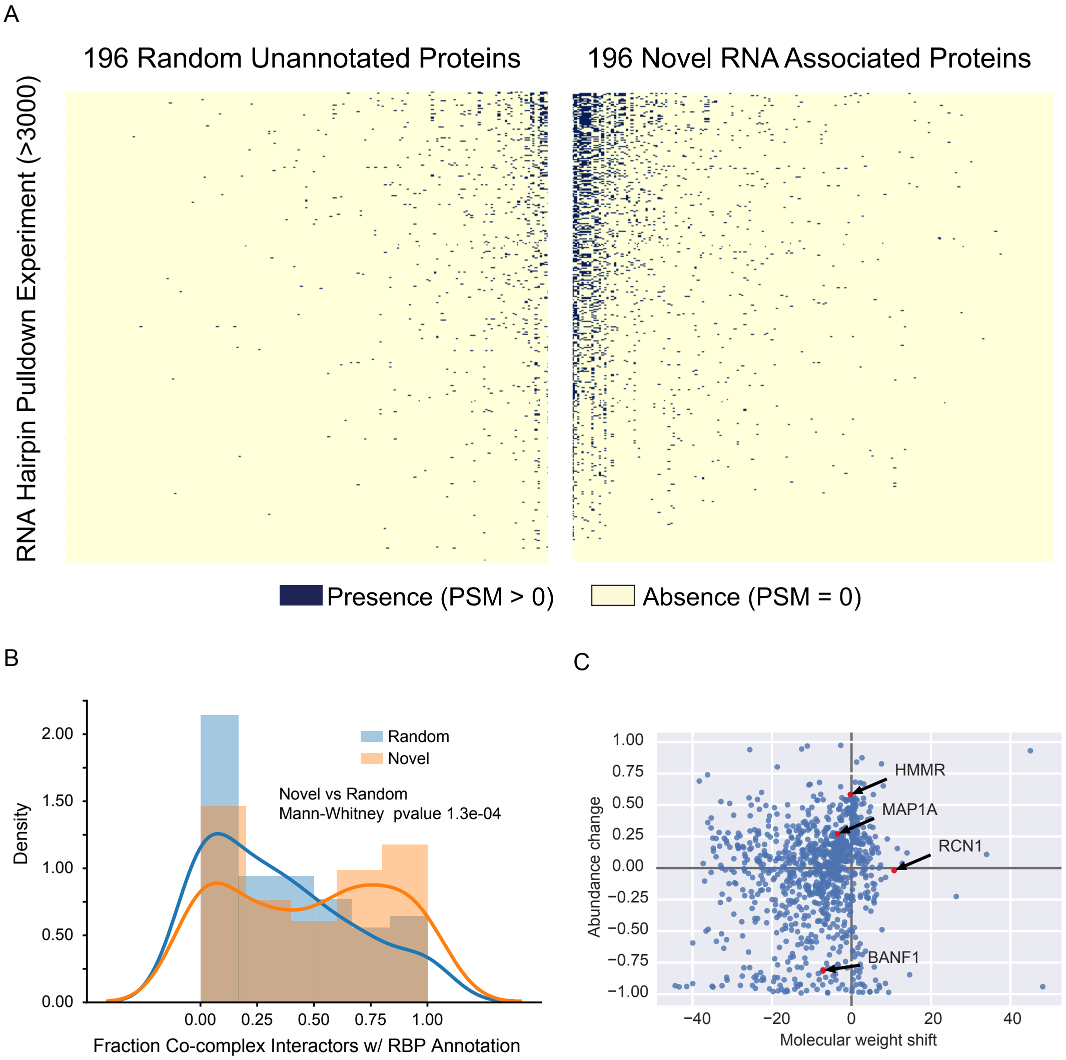

Figure S6: Previously uncharacterized RNA-associated proteins identified by DIF-FRAC identified in RNA hairpin pulldown experiments. Related to Figure 4. (A) Enrichment of presence of 196 novel RNA-associated proteins identified by DIF-FRAC in RNA hairpin pulldowns compared to randomly selected proteins. (B) Distribution of co-complex protein interaction partners of novel RNA-associated proteins with RNA-binding protein annotations. Distribution of novel RNA-associated proteins is right shifted compared to random proteins suggesting they are more likely to interact with previously identified RNA-binding proteins. (C) Analysis of DIF-FRAC shift types of RNA-associated proteins. Upon RNase A treatment we observe different types of changes to protein elution profiles (see Figure 1H and Figure 4). Each point in the graph represents one RNA-associated protein. Molecular weight shift is the weighted average difference between control and RNase A treated profiles, where a negative value (left side of graph) represents lower molecular weight elution upon treatment and positive value (right side of graph) represents gain in molecular weight (see Methods for calculation). Abundance change is the normalized change in observed abundance upon RNase A treatment. A positive value (top of graph) represents gain in solubility and a negative value (bottom of graph) represents loss in solubility. Examples from Figure 4 are annotated.

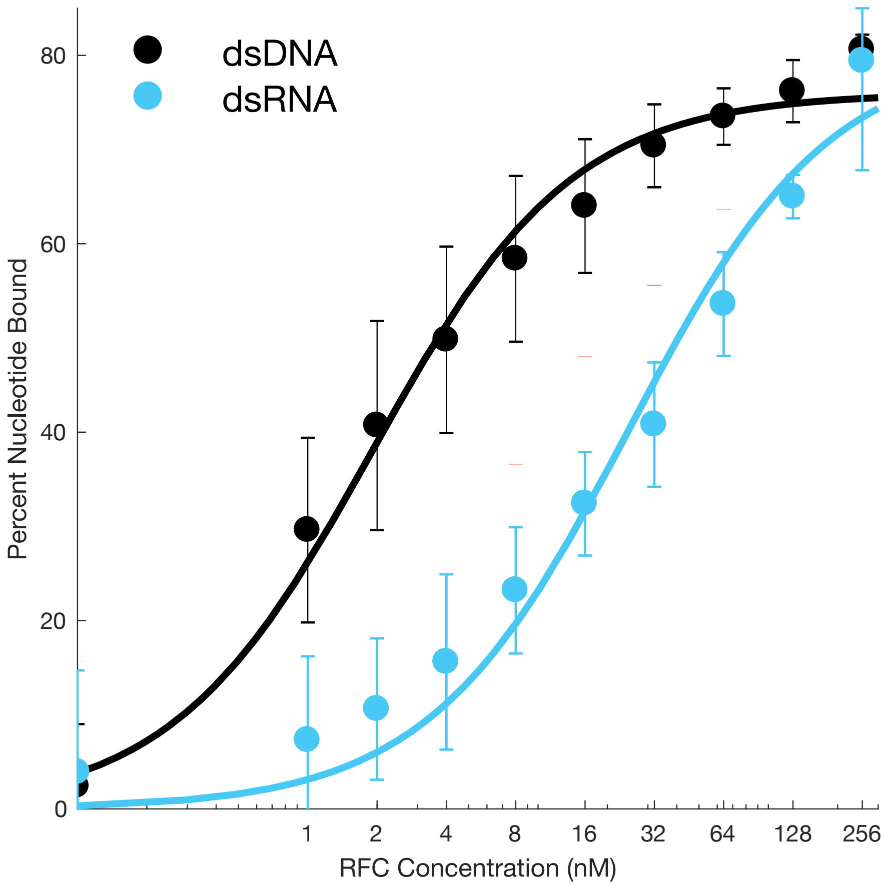

Figure S7: Affinity of nucleic acid for the *S. cerevisiae* RFC complex. Related to Figure 5. Binding curves from electromorphic mobility shift assays (EMSA) of various concentrations of purified *S. cerevisiae* RFC mixed with 1 nM ^{32}P-labeled oligonucleotides. Data was fit to a hyperbolic equation (solid line). The calculated k_D + 95% CI is 1.9 ± 0.5 nM for dsDNA (black), and 25 ± 11 nM for dsRNA (blue). Error bars denote standard deviation.

Table S3: Top 15 unannotated RNA-associated proteins identified by DIF-FRAC. Related to Figure 4.

	Gene Name	Protein	Function	Soluble without RNA? $	Disease links #	DIF-FRAC score/ p-value (5 % FDR)	DIF-FRAC plot
1.	BANF1	Barrier-to-autointegration factor	Chromatin organization	No	Progeria syndrome	6.17E-45	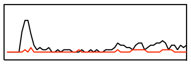
2.	RCN1	Reticulocalbin-1	Calcium binding<br>Secretory pathway<br>Stress Response	No	Amyloid formation<br>Hepatocellular carcinoma	4.97E-41	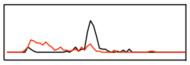
3.	MAP1A	Microtubule-associated protein 1A	Microtubule assembly<br>Structural protein	Yes	Hearing loss	1.38E-39	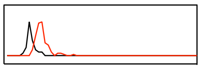
4.	NOMO3	Nodal modulator 3	Carbohydrate binding	Yes	N/A	2.29E-32	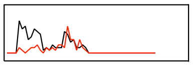
5.	ACTA2	Actin, aortic smooth muscle	Muscle protein	No	Vascular diseases	7.11E-31	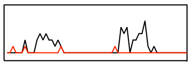
6.	RSBN1L	Round spermatid basic protein 1-like protein	N/A	No	N/A	1.45E-27	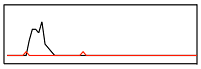
7.	NIPSNAP1	Protein NipSnap homolog 1	Neurotransmitter binding	Yes	N/A	1.92E-24	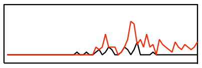
8.	CLSPN	Claspin	DNA binding<br>DNA replication	No	N/A	2.79E-22	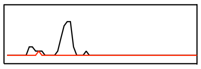
9.	U2AF1L5	Splicing factor U2AF 35 kDa subunit-like protein	RNA binding (by similarity)	No	N/A	3.76E-22	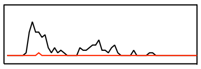
10.	MORF4L2	Mortality factor 4-like protein 2	Chromatin regulator	Yes	N/A	9.82E-22	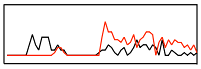
11.	HMMR	Hyaluronan mediated motility receptor	Hyaluronic acid binding	Yes	Breast cancer	2.04E-17	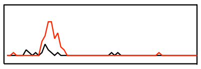
12.	PTGES2	Prostaglandin E synthase 2	Isomerase	Yes	Type 2 diabetes	2.52E-17	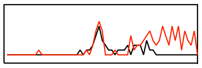
13.	WNK2	Serine/threonine-protein kinase WNK2	Serine/threonine-protein kinase	Yes	N/A	4.14E-17	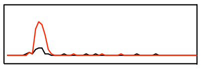
14.	MARK3	MAP/microtubule affinity-regulating kinase 3	Serine/threonine-protein kinase	Yes	Pancreas carcinogenesis	1.40E-16	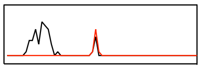
15.	ALOX5	Arachidonate 5-lipoxygenase	Leukotriene biosynthesis	Yes	Asthma	1.21E-15	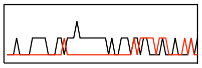

$ Insolubility in the absence of RNA is inferred by an increase in elution volume/ molecular weight of the protein upon RNA digestion, or a complete disappearance of signal. This is consistent with the RNA-associated protein being solubilized by RNA, as suggested by Maharana et al. (Maharana et al., 2018)
# Annotations from UniProt (The UniProt, 2017) and/ or OMIM (https://omim.org/)
